# Health, Wellness, and Place Attachment During and Post Health Pandemics

**DOI:** 10.3389/fpsyg.2020.573220

**Published:** 2020-11-26

**Authors:** Salman Majeed, Haywantee Ramkissoon

**Affiliations:** ^1^Department of Marketing, College of Management, Shenzhen University, Shenzhen, China; ^2^School of Business and Economics, Faculty of Biosciences, Fisheries and Economics, UiT The Arctic University of Norway, Tromsø, Norway; ^3^Derby Business School, College of Business, Law and Social Sciences, University of Derby, Derby, United Kingdom; ^4^College of Business and Economics, Johannesburg Business School, University of Johannesburg, Johannesburg, South Africa

**Keywords:** therapeutic landscapes, health, wellness, tourism, place attachment, COVID-19

## Abstract

Therapeutic landscapes encapsulate healing and recovery notions in natural and built environmental settings. Tourists’ perceptions determine their decision making of health and wellness tourism consumption. Researchers struggle with the conceptualization of the term ‘therapeutic landscapes’ across disciplines. Drawing on extant literature searched in nine databases, this scoping review identifies different dimensions of therapeutic landscapes. Out of identified 178 literature sources, 124 met the inclusion criteria of identified keywords. We review the contribution and the potential of environmental psychology in understanding tourist behavior to promote health and wellness tourism destinations in a post COVID-19 context. We develop and propose a conceptual framework comprising: (1) perceived goodness of therapeutic landscapes, (2) health and wellness consumption, (3) COVID-19 pandemic perceived health and wellness risk, (4) place attachment, and (5) re-visitation. We propose measurement scales and discuss implications and major issues in the immediate and post the COVID-19 pandemic to inform future research.

## Introduction

Drawing on theories of humanism and structural and cultural ecology, Gesler (1992) introduced the concept of therapeutic landscapes and uncovered the healing and recovery coordination between place and environment (Gesler, 2003). Therapeutic landscapes are referred to (1) facilities that offer conventional western medical treatments and alternative health treatments, such as naturopathy, cupping therapy, art therapy, mud therapy, etc. (Williams, 1998; Smith, 2015; Majeed et al., 2017a, 2019a), (2) built attractions, e.g., spa hotels, hospitals, community gardens, etc. (Oster et al., 2011; Adongo et al., 2017; Majeed et al., 2018; Townsend et al., 2018; Ramkissoon, 2020), and (3) travel destinations with natural features (Palka, 1999) which provide restorative experiences alongside the feeling of comfort and gratitude to individuals seeking health and well-being (Hansen et al., 2017). Research on therapeutic landscapes continues to attract the attention of scholars across different fields including urban planning for landscape development (Rodiek and Fried, 2005), health geography (Smyth, 2005), nursing (Kennedy et al., 2004), psychology (Coghlan, 2015), holistic medicine (Williams, 1998), traditional and complementary medicine (Majeed et al., 2017a), medical tourism (Buzinde and Yarnal, 2012; Majeed and Lu, 2017; Majeed et al., 2018), public health (Love et al., 2012; Williams, 2014), and religion (Agyekum and Newbold, 2016).

Therapeutic landscapes are a combination of physical and built environments with human perceptions and social interactions that interact with each other to produce a sense of healing (Gesler, 2003; English et al., 2008) which generates happiness and contributes to the overall quality of life. Life satisfaction, happiness, and quality of life notions are noted as the core constructs of health and well-being (Smith, 2015; Ramkissoon, 2020) or sometimes just well-being. Researchers also examine the contribution of healing places to human health and well-being, which are noted in terms of connections of mind, body, and soul (Townsend et al., 2018; Majeed et al., 2019a). Wellness and spa tourism is becoming increasingly popular with destination marketers offering a range of products including therapeutic landscapes (Wakefield and McMullan, 2005; Zhou et al., 2017). These retreats often have state-of-the-art environmental settings and are well equipped with staff ranging from nutritionists, sports physiologists, and naturopaths whose combined expertise assist in co-creating healing and wellness experiences with customers (Ramkissoon, 2014). Meeting individuals’ expectations and perceptions give a feeling of satisfaction and happiness which are orthogonal to quality of life (Ramkissoon et al., 2018; Majeed et al., 2020a,b).

Researchers have examined the traveling trends of people across the globe with hopes and expectations to find different health and well-being treatments (Smith, 2015; Majeed et al., 2017a, 2018, 2019a; Ramkissoon et al., 2018). Individuals perceive that traveling to destinations with abundant therapeutic landscapes, such as health resorts, gardens, meadows, lakes, and wellness retreat centers, improves their health and well-being. Scholars note the increasing trends in health (medical) and wellness tourism that allow tourists to rest and relax or spend time at therapeutic landscapes after surgery for recuperation (Ramkissoon et al., 2013a; Majeed et al., 2018; Kaspar et al., 2019). People in the Baltic States report they search to improve their health and well-being by enjoying the spa, being at the beach, and exploring forests (Smith, 2015). Some scholars mention the healing properties of the sea, trees, and fossil-based treatments at destinations (Dryglas and Salamaga, 2018).

Therapeutic landscapes have attracted significant attention from scholars and practitioners. However, research examining the interplay of relationships between visits to therapeutic landscapes, place attachment, and re-visit intentions to health and wellness tourism destinations is still at an infancy stage. The premise of this scoping review is to explore the associations between these constructs. We further explore how the global health crisis COVID-19 may influence health tourism and wellness tourism.

The scoping review aims to examine, first, how do people’s perceived goodness of therapeutic landscapes influence their decisions to visit a destination’s therapeutic landscapes? Second, what are the associations between people’s visits to wellness and health tourism destinations and place attachment? Third, how does a global health crisis impact on visitors’ re-visitation to health and wellness tourism destinations? We use the present global pandemic COVID-19 as the context. Our objective is to develop and propose a conceptual framework and hypotheses to present the associations between perceived goodness of therapeutic landscapes, health and wellness tourism consumption, place attachment, and re-visitation to therapeutic landscapes and the potential influence of COVID-19 perceived risk on these relationships. This has important implications for future research and practice across different disciplines. We examine the varying concepts of therapeutic landscapes into an integrated research framework that may provide insights to scholars and practitioners in the service industries and assist in the effective management of therapeutic landscapes and in their marketing efforts to attract more national and global visitors.

We followed the scoping review technique (Arksey and O’Malley, 2005) to achieve scoping review objectives. Our scoping review of the published literature between 2000 and 2020 addresses the woven connections of sub-constructs of therapeutic landscapes that influence people’s perceptions of therapeutic landscapes to improve their health and well-being that may further impact on their place attachment.

## Materials and Methods

An initial search was conducted using relevant keywords, such as therapeutic landscapes, health and place, visits to therapeutic landscape, landscapes, health and well-being, and therapeutic landscapes and disasters, in three electronic databases (Google Scholar, Scopus, and Web of Science). To expand the literature searching strategy in a focused way, the reference mining technique was adopted. Reference mining refers to analyzing the references of already searched literature. It allows researchers to find more relevant literature sources quickly from already searched literature in the context of the review (Majeed et al., 2017a). Relevant keywords were searched in nine databases, including Google Scholar, PubMed, Web of Science, MDPI, Taylor and Francis, Scopus, Springer Nature, Elsevier, and Sage, to search articles and books published between 2000 and 2020.

The title, abstract, and keywords of all identified literature were reviewed by the researchers. The difference of opinions on whether to conduct a full or partial review at the initial stage was resolved within the weekly team meetings of the researchers. An in-depth review of the literature was conducted based on the initial findings of the title, abstract, and keywords. We organized, charted, and collated the extracted data of 124 literature sources in a commonly accessible online spreadsheet (see Appendix 1 for full literature spreadsheet). Researchers recorded the emerging themes from the reviewed sources, i.e., title, research approach, and conclusions in brief on the online spreadsheet accessible by the researchers.

**FIGURE 1:**
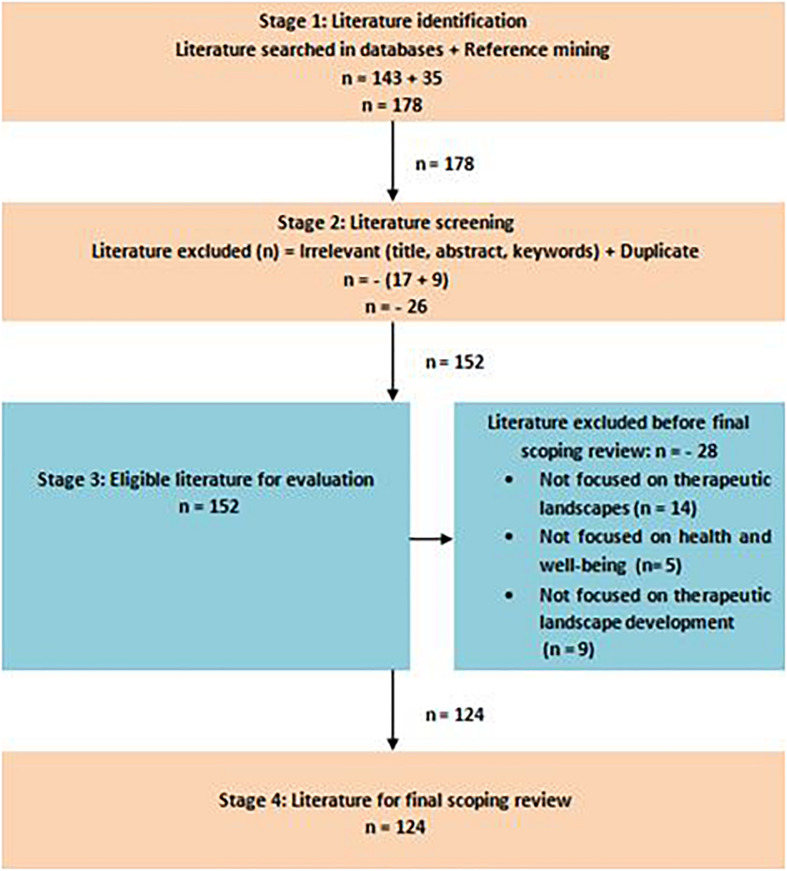
PRISMA flowchart.

A total of 124 literature sources were retained after screening and determining the eligibility of the gathered literature (see Figure 1). A list of searched literature with main notes and extracted themes is presented in Appendix 2.

## Results

Table 1 shows that searched literature sources belong to different research domains. The majority of searched literature belongs to the field of geography (30.65%) followed by health and wellness tourism (25.81%). Table 2 shows that the majority of searched literature was journal articles (86.29%) followed by book chapters (8.06%) and books (5.65%). Findings of the scoping review are summarized in the next sections.

**TABLE 1:** Research domains of reviewed sources.

**Disciplines**	**Frequency**	**Percentage**	**Cumulative percentage**
Health and wellness tourism	32	25.81	25.81
Environmental Psychology	13	10.48	36.29
Medical and alternative health practices	11	8.87	45.16
Crises management	19	15.32	60.48
Public Health	11	8.87	69.35
Geography	38	30.65	100
Total	124	100	

**TABLE 2:** Distribution of reviewed sources.

**Type of source**	**Frequency**	**Percentage**	**Cumulative percentage**
Journal articles	107	86.29	86.29
Books	7	5.65	91.94
Book chapters	10	8.06	100
Total	124	100	

### Perceived Goodness of Therapeutic Landscapes

Perceived goodness of therapeutic landscapes involves multi-dimensional concepts in its breadth and depth (Pitt, 2014; Adongo et al., 2017) calling for more research into the operationalization of the term. Our scoping review sheds further light on the operationalization of perceived goodness of therapeutic landscapes. The notion of individuals’ feelings of health, healing, and recovery in tandem with attitudes toward therapeutic landscapes is interpreted as perceived goodness of therapeutic landscapes (Völker and Kistemann, 2011). The resources of a destination’s therapeutic landscapes, whether natural or built, shape visitors’ perceptions of feeling well and living well (Bolten and Barbiero, 2020). Environmental pollution is a pressing concern for people’s health as it threatens the ecological infrastructure of a destination (Wang X. et al., 2018).

Our analysis reflects that landscape resources, such as clean water, blue sky, and pollution-free environment, favorably influences people’s perceived goodness of therapeutic landscapes that may contribute to their optimal health and well-being (Steinwender et al., 2008). The quality of the environment determines the quality of human health and the healing process. Scholars across disciplines including geography, public health, and tourism present the concept of therapeutic landscapes in terms of places offering hygienic food, clean hospitals, necessary medical interventions, wellness treatment, clean accommodation, beaches, fresh air, and natural landscapes to boost the anatomical connection of the mind, body, and soul (Majeed et al., 2018; Ramkissoon and Sowamber, 2018; Townsend et al., 2018). Natural resources, however, may also pose potential threats to individuals’ health and well-being because of the length of time consumed in therapeutic landscapes (Adongo et al., 2017).

#### Natural Landscapes

Therapeutic landscapes are categorized as blue space (Britton et al., 2020), e.g., beach, river, lake, and spa, and green space, e.g., gardens and groves (Foley and Kistemann, 2015; Bell et al., 2017; Zhou et al., 2017). People travel to therapeutic landscapes to find blue and green space in warm climates in search of peaceful and distinctive environments suitable to their mind and body (Zhou et al., 2017). Green space often gives a sense of meaningful purpose in life (Ramkissoon et al., 2013c) and enhances mood and energy (Hansen et al., 2017). Evidence shows that people travel to parks to find healing, recovery, and confidence; for some, these places provide spectacular landscapes and experiences (Hall and Page, 2002; Ramkissoon et al., 2012). For example, people express they could feel the healing and recovery in the Denali National Park of Alaska and considers it as an extraordinary therapeutic landscape (Palka, 1999).

Blue and green spaces involve the phenomenon of exposure to natural stimuli; researchers argue it is part of nature therapy due to their preventive medical effects on people’s psychological and physical health (Hassan and Ramkissoon, 2020), such as reducing the risk of stress and mood disorders, hypertension, and heart attack and increasing the feeling of comfort and gratitude (Hansen et al., 2017). Despite blue space and green space notions, people also travel to deserts and consider the yellow space as an important natural therapeutic landscape promoting their health and well-being. Turpan city desert in China is an interesting destination for seekers of health and wellness; they engage in sand bathing to seek a cure for their respective illness (Wang K. et al., 2018). Drawing on the above, natural landscape with blue, green, and yellow space may influence individuals’ perceived goodness of therapeutic landscapes. People attempt to travel to destinations with a variety of therapeutic landscapes to promote their health and well-being.

#### Built Landscapes

People travel to community places such as community gardens and hospitals (if needed) to promote their well-being and quality of life (Milligan et al., 2004; English et al., 2008; Love et al., 2012). Traditional and complementary medicine also supports the healing and recovery impacts of built therapeutic landscapes, such as spiritual retreats and spa town (Oster et al., 2011; Majeed et al., 2017a). Spa hotels in Estonia are known for the best healing and recovery treatments with medical spa, diet, and rehabilitation (Smith, 2015). Holy wells, mosques, and churches are also known as built landscapes for healing and recovery of the mind, body, and soul combined with spirituality (Foley, 2010; Agyekum and Newbold, 2016). Many recovery programs, such as alcohol prevention and recovery, are developed at built landscapes with gardens, medical assistance, and physical exercises to improve the health and well-being of people (Wilton and DeVerteuil, 2006; Love et al., 2012). Built landscapes may provide physical settings allowing people to interact with nature being brought to them, contributing to their wellness and enhancing their overall life satisfaction. Built landscapes at wellness destinations may influence individuals’ perceived goodness of therapeutic landscapes providing satisfaction, happiness, feeling of comfort, a sense of achievement, healing, and recovery, thus promoting visitors’ health and well-being (English et al., 2008; Zhou et al., 2017).

#### Landscape Design

Literature on therapeutic landscape shows that landscape design is an important element of perceived goodness of therapeutic landscapes. Water holds special importance in the concept of landscape design (Asakawa et al., 2004). Natural scenery, such as calm aquatic scenes and gently curving banks, positively influences human feelings of healing and recovery (Asakawa et al., 2004; Steinwender et al., 2008; White et al., 2010). Landscape design with waterscapes integrates aesthetic and ecological characteristics to amuse and relax humans for their health and well-being in urban areas (Karmanov and Hamel, 2008). The Spa Town in Buxton in the peak district in the United Kingdom is known to attract visitors to its water festivals (Maxfield and Wiltshier, 2019). Landscape design helps to satisfy individuals with cheerfulness and promote health and well-being (Myers, 2020). The structural biodiversity of therapeutic landscapes, such as trees, vegetation, architecture, history, symbols, wildlife, and water, gives the feeling of healing, recovery to individuals traveling for health, and well-being (Yamashita, 2002; Townsend et al., 2018). Landscape design with distinctive features fuels individuals’ perceived goodness of therapeutic landscapes which can enhance place attachment.

#### Therapeutic Networks

Therapeutic networks are external supporting factors to ensure individuals’ health and well-being at healing and recovery places. Therapeutic networks sometimes function at a micro-level, including doctors, paramedical staff, and practitioners of traditional and complementary medicine (Smyth, 2005; Majeed and Lu, 2017; Majeed et al., 2018). Therapeutic networks also function at a macro-level, including hospitals, asylums, gardens, towns, regions (Andrews, 2004; Andrews et al., 2005; English et al., 2008; Majeed et al., 2017a), and supporting service providers, such as the hospitality industry. For instance, the accommodation, food and beverage suppliers, and shopping stores provide the necessities to assist health and wellness visitors (Smyth, 2005; Majeed and Lu, 2017; Majeed et al., 2018).

Scholars discuss the concept of therapeutic mobilities in terms of supporting therapeutic factors, such as pharmaceuticals, gifts, paramedical staff, information, and narratives, which unfold through the network of regions and markets to develop an infrastructure to improve the therapeutic capacities of a destination’s landscape (Kaspar et al., 2019). Such human and non-human elements exist at the therapeutic landscapes to assist individuals seeking health and well-being benefits. Religious centers, resorts, spa towns, caves, hot springs, bath houses (Dryglas and Salamaga, 2018), drug-treatment centers, alcohol prevention facilities, neighborhood treatment settings, and home-alike health centers (Love et al., 2012) coordinate together to deliver healing and recovery services to individuals. These healing and recovery service providers and therapeutic service actors (see Kaspar et al., 2019) further extend the understanding of a broader infrastructure of therapeutic networks that may influence individuals’ perceived goodness of therapeutic landscapes.

### Holistic Medicine and Place

Evidence suggests that individuals’ health and well-being are linked to the environment, both natural and built, for healing and recovery (Hall and Page, 2002; Steinwender et al., 2008; Adongo et al., 2017; Majeed et al., 2018). Perceived goodness of therapeutic landscapes encapsulates the enduring healing of physical and mental health with a combination of treatment of illness and landscape (Gesler, 1992, 2003). The associated combination of place and treatment for illness is documented in health geography, medical tourism, and wellness tourism literature (Majeed et al., 2017a). The notion of holistic medicine involves place-based treatments with traditional and complementary medicine, such as naturopathy, homeopathy, spa, cupping therapy, art therapy, touch therapy, and mud therapy, in botanical gardens and beaches (Williams, 1998; Majeed et al., 2017a, 2019a). Therapeutic landscapes often help to maintain health with the healing effects of place, including blue and green environmental settings (Adongo et al., 2017). Activities at therapeutic places help to balance the biodiversity of the mind, body, and soul (Smith and Puczkó, 2014). Place-based wellness treatments, such as mud therapy (especially with sea mud), Ayurveda, and acupuncture, may positively influence wellness (e.g., treatment for beauty enhancement) and health (e.g., treatment of underlying health conditions) (Smith, 2015) and in turn promote place attachment.

Individuals’ preferences for travel and optimal well-being are noted in terms of lifestyle treatments, such as cosmetic surgery or plastic and reconstructive surgery, to perform better in their everyday matters (Majeed et al., 2019b). Soft environments in healing places, e.g., hospitals with polite medics and facilities to accommodate patients’ family and friends, are considered conducive to individuals’ healing and recovery (Williams, 1998). Singapore General Hospital and Mount Elizabeth Hospital are examples of adopting a holistic medicine approach showcasing a soft environment to patients for them to relax and bring a feeling of good health for quick recovery. Associations between human feelings, expectations, and perceptions of therapeutic landscapes play an important role in physical and mental health which is referred to as ‘mind landscape.’ This is because the cohesion of human psychological filters with the surrounding environment and visualization produces imagery to sketch the mind landscape (Williams, 1998).

Holistic medicine offers treatments which are place-based. Williams (1998) and Cremers (2020) documents that sense of place points to the feelings of family life, aesthetics, identity, and security which transcends over a passage of time with a longer stay at a particular place. Ramkissoon et al. (2013c) investigate place attachment with sub-dimensions of place dependence, place identity, place affect, and place social bonding in a national park setting in Australia. Individuals with high place affect experience a sense of psychological well-being (Ramkissoon et al., 2013b). Place attachment in nature-based settings also positively influences visitors’ quality-of-life (Brown and Raymond, 2007; Ramkissoon et al., 2018). A strong sense of attachment to the place may lead to enduring therapeutic effects on individuals’ health and well-being. Such concepts are deeply linked to therapeutic landscapes with natural and built environments (Gatrell, 2013). For example, temples, mosques, churches, and asylums belong to different religious beliefs; people visiting religious places may assign positive meanings to the place leading to healing and recovery.

The association of a place with the health and well-being enhancement is referred to as psychological rootedness (Gesler, 1992) in literature. Some evidence suggests individuals travel to remote reputable therapeutic places for optimal health and well-being (English et al., 2008). Ancient sites, e.g., Epidaurus in Greece, the hot springs of South Dakota, and spiritual retreats in Lourdes, France, are some examples to illustrate the links between visitors’ health and wellness and place attachment (English et al., 2008).

### Health, Wellness Tourism, and Place Attachment

Improvement in individuals’ physical and mental health may ensure life satisfaction, happiness, and quality of life (Gatrell, 2013; Majeed et al., 2019a). Studies on human perceptions and behaviors note individuals’ may experience positive feelings due to favorable trade-offs between their expectations and perceptions (Liamputtong and Suwankhong, 2015; Majeed and Lu, 2016; Majeed et al., 2020a,b; Saqib et al., 2020; Xue et al., 2020). Health and well-being are determined by the psychological interplay of individuals’ expectations and perceptions of blue and green spaces (e.g., Völker and Kistemann, 2011; Ramkissoon, 2020), which further impact their travel decisions (Ramkissoon and Mavondo, 2015; Smith, 2015; Majeed et al., 2020b).

Attention restoration theory presents that individuals’ directed and fascinated attention determines their actions to overcome complex impressions of everyday life (Stigsdotter et al., 2010). Individuals’ attention exhausts quickly in the absence of appropriate recovery opportunities. People recover well in a comfortable environment. Restorative environments include traveling to places where the feeling of connectedness (human–environment bond) (Ramkissoon et al., 2013b), compatibility (human needs and supporting resources at the host place) (Nunkoo and Ramkissoon, 2016), and fascination (attraction and uniqueness of host place) (Jiang et al., 2017) are fulfilled (Stigsdotter et al., 2010). The aesthetic-affective theory presents that natural environments contain the stimulus that restores individuals’ more positive views of himself/herself and his/her capacities with decreased stress (Stigsdotter et al., 2010).

Individuals’ visits to destinations’ therapeutic landscapes are determined by their intrinsic motivations which originate through the interplay of perceptual filters to find health and well-being benefits (Jeuring and Becken, 2013; Liamputtong and Suwankhong, 2015). The theory of therapeutic landscapes suggests that human health and well-being is a place-based concept (Gesler, 1992), suggesting people find healing benefits in connection to place (e.g., land, sea, forest, and cities) and develop an emotional bond with these environmental settings (Ramkissoon et al., 2013a; Ramkissoon et al., 2013c). Emotions have a strong influence on human behaviors and develop an emotional attachment with a person, object, and environment (Majeed et al., 2017b). This emotional bond between people and place is commonly known as place attachment.

Place attachment originates from attachment theory (Bowlby, 1982) depicting the mother–infant bond. This relationship expands to include other relationships with social, natural, and built environments, including therapeutic landscapes. Ramkissoon et al. (2013a, b) argue that individuals develop a sense of place dependence, place identity, place affect, and place social bonding as their relationships expand. Individuals may be place-dependent on therapeutic landscapes as these settings serve their functional purpose (Stokols and Shumaker, 1982), and the visitors might not want to substitute this place for another. People may also feel a sense of identity with therapeutic landscapes due to their distinctive features (Proshansky, 1978; Ramkissoon and Mavondo, 2015).

Individuals may form social connections (Ramkissoon et al., 2018) at therapeutic landscapes which can assist greatly in their mental well-being. People coming together may collectively create meanings (Nye and Hargreaves, 2010) at the therapeutic landscapes which promote relaxation and other mental and physical health benefits (Ramkissoon et al., 2013b). Some evidence show associations of natural and built therapeutic landscapes help to alleviate individuals’ stress and promote a sense of health and well-being. This may in turn promote place attachment to therapeutic settings (Ramkissoon and Mavondo, 2017). The combined philosophies of the attention restorative theory and the aesthetic-affective theory, therapeutic landscape theory, and place attachment theory provide theoretical support for our proposed theoretical model.

### Global Health Crisis COVID-19

People’s feelings of the likelihood of being a victim of COVID-19 have fueled perceived risk in travel which may impact negatively future travel decision-making. Health and wellness tourism destinations are likely to be highly impacted. Despite the health-enhancement attractions of visits to therapeutic landscapes, perceived risk of the likelihood of a health-related crisis, such as COVID-19, can disrupt the balance of individuals’ favorable perceptions, visit/re-visit intentions (Novelli et al., 2018).

The Greek word “krisis” coined the term “crisis” which means decision, choice, or judgment. Different research contexts interpret “crisis” differently. Scholars note “crisis” in terms of ambiguous cause and effect that threatens organizational viability, reputation, and individuals’ lives with the belief of quick resolution (Pearson and Clair, 1998). Destination crisis is also noted in terms of events that lead to adverse situations (Seymour and Moore, 2000). Improper understanding of destination crisis may undermine consumers’ confidence in the service provider’s competence for business and society (Laws and Prideaux, 2005). COVID-19 pandemic may impact individuals’ visits to health and wellness destinations. Scholars note that health crises in the past, such as Middle East respiratory syndrome coronavirus (MERs-Cov) in the Middle East, severe acute respiratory syndrome (SARs) in South East Asia, EBOLA in Africa, foot and mouth disease in the United Kingdom, and influenza pandemic in Mexico, increased tourists’ doubts on the credibility of epidemic-hit destinations and discouraged them to visit or re-visit such destinations for health and well-being benefits (Pine and McKercher, 2004; Wilder-Smith, 2006; Mao et al., 2010; Pavli et al., 2014; Baker, 2015; Novelli et al., 2018).

Risk is noted as an important factor which shapes human decision making (Rittichainuwat and Chakraborty, 2012; Ramkissoon, 2018). Different people perceive traveling risks differently (Aro et al., 2009). Individuals’ visits to destinations’ therapeutic landscapes are tightly linked to health, healing, recovery, and longevity. Scholars note that health crises exert long-lasting negative impacts on re-visits to destination’s therapeutic landscapes (Tse, 2006). Overall life satisfaction is important for the health and well-being of our people (Townsend et al., 2018; Majeed et al., 2019a, 2020b). Individuals’ visits to destination’s therapeutic landscapes and resultant health and well-being benefits are associated with experiences derived from healthy places; epidemic-hit places may discourage re-visitation. COVID-19 is likely to have a huge impact on the health and wellness tourism consumers and providers.

## Discussion, Conceptual Framework, and Contribution

Previous research examined different aspects of therapeutic landscapes across different research contexts (Smyth, 2005; Love et al., 2012; Coghlan, 2015; Majeed et al., 2017a, 2018). Our review of individuals’ perceived goodness of therapeutic landscapes reflects the core constituents of therapeutic landscapes which may impact health and well-being tourist consumption which may in turn influence place attachment. We further explore perceived risk of COVID-19 and its associations with the interacting phenomena of therapeutic landscapes promoting health and wellness tourism.

**FIGURE 2:**
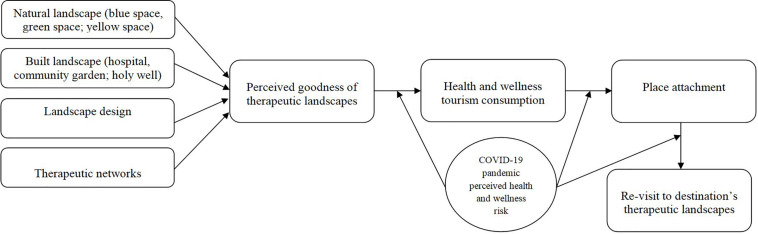
Theoretical framework.

Perceived goodness of therapeutic landscapes involves different service platforms in its breadth and depth to ensure individuals’ health tourism, wellness tourism, place attachment, and re-visits to destinations’ therapeutic landscapes. Individuals seek natural landscapes, built landscapes, good landscape designs, and therapeutic networks for managing and improving their health and well-being. They may get emotionally attached to such places encouraging re-visits. However, this is likely to be disrupted by the current COVID-19 public health crisis. COVID-19 pandemic perceived health risk may exert its negative influence on the respective relationships between perceived goodness of therapeutic landscapes and health tourism, wellness tourism, place attachment, and re-visit to destination’s therapeutic landscapes. Based on our scoping review of extant literature, we develop an integrative theoretical framework (Figure 2).

Figure 2 presents that visitors’ perceived goodness of therapeutic landscapes positively impacts health and wellness tourism consumption and place attachment. Further, our model posits that visitors’ place attachment positively influences their re-visit to the destination. The relationship between perceived goodness of therapeutic landscapes, health and wellness tourism consumption, place attachment, and re-visit to destination’s therapeutic landscapes is negatively impacted by COVID-19 pandemic perceived health and wellness risk.

A total of 40 items are borrowed from extant literature to measure the proposed constructs in this scoping review (see Table 3), i.e., perceived goodness of therapeutic landscapes (six items), wellness tourism (eight items), health tourism (six items), place attachment (nine items), re-visit to destination (five items), and COVID-19 pandemic perceived health risk (six items). Hinkin et al. (1997) present a seven-step process for scale construction. We developed scale items for each construct as presented in Table 3, which were needed to support idea generation and to start the process of developing a new scale for therapeutic landscapes and health and wellness tourism consumption in the context of health pandemics, such as COVID-19. We note that the content adequacy assessment with factor analytical technique alongside a comparison of mean scores is needed (Hinkin et al., 1997). It is suggested to pre-test this new scale with an adequate sample of health and wellness tourism consumers. A minimum sample size of 100 is suggested on a seven-point Likert scale to examine discriminant, convergent, and criterion-related validity (Hinkin et al., 1997). We suggest researchers employ an exploratory factor analysis for the scale measurement.

**TABLE 3:** Items and scales included from different searches.

**Perceived goodness of therapeutic landscapes**	**Adapted from**
Therapeutic landscapes with natural resources will help to find better mental and physical health.	Asakawa et al., 2004; Nunkoo and Ramkissoon, 2012; Smith, 2015
Therapeutic landscapes will provide better caring environment than expected.	Smith, 2015
Therapeutic landscapes will help to strengthen immune system of the body.	Kennedy et al., 2004
The environment of therapeutic landscapes will be good for rest, sleep, and rehabilitation.	Kennedy et al., 2004
Therapeutic landscapes with a supporting network of people and services will help in quick healing and recovery.	Kennedy et al., 2004; Rodiek and Fried, 2005; Zhou et al., 2017
Therapeutic landscapes will provide aesthetic services (e.g., alternative treatments, diet and exercise plan, etc.).	Smith, 2015
**Wellness tourism**	
Visiting and staying at a destination’s therapeutic landscapes helps me perform well in my daily life activities.	Kennedy et al., 2004; Rodiek and Fried, 2005
Visiting and staying at a destination’s therapeutic landscapes allows me to spend happy time with other people.	Kennedy et al., 2004; Rodiek and Fried, 2005
Visiting and staying at a destination’s therapeutic landscapes helps me to feel refreshing.	Kennedy et al., 2004
Time spent at a destination’s therapeutic landscapes makes my life enjoyable.	Kennedy et al., 2004; Rodiek and Fried, 2005
Visiting and staying at a destination’s therapeutic landscapes helps me to feel positive energy.	Kennedy et al., 2004
Visiting a destination’s therapeutic landscapes helps me to find peace of mind, body, and soul.	Rodiek and Fried, 2005
Staying at a destination’s therapeutic landscapes helps me to maintain a good mood.	Kennedy et al., 2004; Rodiek and Fried, 2005
Visiting and staying at a destination’s therapeutic landscapes helps me to feel the renewal, restoration, and rejuvenation.	Larsen et al., 2009
**Health tourism**	
Visiting and staying at a destination’s therapeutic landscapes helps me to live longer.	Kennedy et al., 2004; Rodiek and Fried, 2005
Visiting and staying at a destination’s therapeutic landscapes helps me to improve my physical health.	Kennedy et al., 2004; Larsen et al., 2009
Visiting and staying at a destination’s therapeutic landscapes helps me to improve my mental health.	Larsen et al., 2009
Staying at a destination’s therapeutic landscapes allows me to relax mentally and physically.	Smith, 2015
Visiting and staying at a destination’s therapeutic landscape helps me to find desired health treatments.	Majeed et al., 2018
Visiting and staying at a destination’s therapeutic landscape helps me to find valuable healthcare than the care available in other health facilities/destinations.	Majeed et al., 2018
**Place attachment**	
For the activities that I enjoy most, the settings and facilities provided by this place are the best.	Ramkissoon et al., 2013a,b,c; Ramkissoon and Mavondo, 2015
For what I like to do, I could not imagine anything better than the settings and facilities provided by this place.	Ramkissoon et al., 2013a,b,c; Ramkissoon and Mavondo, 2015
I identify strongly with this place.	Ramkissoon et al., 2013a,b,c; Ramkissoon and Mavondo, 2015
I feel a strong sense of belonging to this place and its settings/facilities.	Ramkissoon et al., 2013a,b,c; Ramkissoon and Mavondo, 2015
This place means a lot to me.	Ramkissoon et al., 2013a,b,c; Ramkissoon and Mavondo, 2015
This place says a lot about who I am.	Ramkissoon et al., 2013a,b,c; Ramkissoon and Mavondo, 2015
I am very attached to this place.	Ramkissoon et al., 2013a,b,c; Ramkissoon and Mavondo, 2015
My friends/family would be disappointed if I were to start visiting other settings and facilities.	Ramkissoon et al., 2013a,b,c; Ramkissoon and Mavondo, 2015
If I were to stop visiting this place, I would lose contact with a number of friends.	Ramkissoon et al., 2013a,b,c; Ramkissoon and Mavondo, 2015
**COVID-19 pandemic perceived health risk**	
I worry that my health might suffer from the occurrence of infectious disease at the host destination.	Law, 2006
I worry that my traveling decision might be affected by the threat of infectious disease at the host destination.	Law, 2006
I worry that I might be exposed to the risk of contagious diseases.	Dolnicar, 2007
I do not worry about the happening of epidemics at the host destination.	Larsen et al., 2009
During traveling to a destination, I constantly worry that something may go wrong.	Larsen et al., 2009
A thermometer to measure fever will help to monitor my health and protect myself from disease (if any).	Rittichainuwat and Chakraborty, 2012
**Revisitation to destination’s therapeutic landscapes**	
I will re-visit a destination’s therapeutic landscapes to find healing places.	English et al., 2008
I will re-visit a destination’s therapeutic landscapes to find refuge for health optimization.	English et al., 2008
I will re-visit a destination’s therapeutic landscape to find like-minded people for exercise and fun.	English et al., 2008; Zhou et al., 2017
I will re-visit a destination’s therapeutic landscapes for healing and recovery than stand-alone facilities.	Majeed et al., 2017a
I will re-visit a destination’s therapeutic landscapes to feel healing power of nature.	English et al., 2008; Larsen et al., 2009

### Theoretical Contributions

Our review is the first to develop and propose a single integrative model exploring associations between perceived goodness of therapeutic landscapes, health and wellness tourism consumption, place attachment, and re-visitation to therapeutic landscapes and the potential influence of perceived risk of the global health pandemic COVID-19 on these relationships. An understanding of therapeutic landscapes from the broader spectrum of healing and recovery elements to promote individuals’ health and well-being, place attachment, and re-visitation to health and wellness tourism destinations remains scant in the literature. Our scoping review shows that the literature on therapeutic landscapes examined different perspectives of healing and recovery places where the focus of research is on one or more aspects of the therapeutic landscape. With a focus on individuals’ perceptions of the goodness of therapeutic landscapes, our review attempts to propose the concept of therapeutic landscapes that is not confined within a particular research domain.

The proposed integrated theoretical framework may serve as a roadmap to develop new theoretical understandings across different disciplines including geography; tourism; marketing and management; psychology; sociology; conventional medical, traditional, and complementary medicine; and public health. The present review makes important contributions to landscape management, public health, place attachment and health tourism, and wellness tourism literature. Our review further contributes to the body of knowledge in understanding the perceived health impacts of COVID-19 on visitors’ travel decision making to health and wellness tourism destinations during and post the COVID-19 global health crisis. Another contribution is the proposed scale measurement items to test the proposed framework.

### Practical Implications

Our review shows that individuals travel to therapeutic landscapes offering a combination of natural and built landscapes, landscape design, holistic medicine, and therapeutic network to promote their health and well-being. Visitors expect to improve their well-being and quality of life (Nunkoo and Ramkissoon, 2011a,b, 2012) with health and wellness tourist offerings (Beirman, 2003; Smith, 2015). Policymakers may draw from our conceptual framework (Figure 2) for the development of therapeutic landscapes with health and wellness attractions matching travelers’ expectations. Bringing nature to people will promote quality of life and healthy nations, hence contributing to the sustainable development goals (Ramkissoon, 2016, 2020). While people’s place attachment to health and wellness tourism destinations may have possibly encouraged re-visitations in a pre-COVID-19 context, the global health crisis is likely to influence visitors’ health risk perceptions and may impact negatively on tourist demand (Pine and McKercher, 2004). Destination marketers and policymakers will need to invest further efforts and resources in promoting destination image and place attachment during and after COVID-19.

The health of people depends on environmental health demanding urgent actions on the protection of our biodiversity (Ramkissoon and Mavondo, 2017; Ramkissoon, 2020). While COVID-19 has threatened human health, failure to address calls to protect therapeutic landscapes will continue to jeopardize both human and environmental health. Stakeholder engagement (Nunkoo and Ramkissoon, 2013; Ramkissoon and Sowamber, 2020) is essential in managing therapeutic landscapes and promoting health and wellness tourism. DMOs, therapeutic service providers, and governments need to collaborate (Hristov and Ramkissoon, 2016; Hristov et al., 2018; Naumov et al., 2020) to develop and implement effective measures to deal with health crises such as COVID-19 and other crises and disasters at the pre-crisis stage, during, and post-crisis stage. This is important to sustain the health and environment sectors, tourism and hospitality businesses, and other niche industries to meet people’s health and well-being needs, hence promoting place attachment and re-visitation. Therapeutic landscapes and more inbound visits may show visitors’ trust (Nunkoo and Ramkissoon, 2012; Nunkoo et al., 2012) in the destination’s therapeutic landscapes and contribute to sustainable economic growth.

### Future Research Directions

We recommend that future researchers test and validate our proposed framework. Scale items presented to measure the constructs under investigation are subject to content adequacy assessment, pre-test questionnaire administration, factor analysis, internal consistency assessment, and construct validity to conclude a final set of scale items for future empirical research. We identified different underlying concepts of therapeutic landscapes and developed an integrative conceptual framework in the ongoing scenario of COVID-19 global health pandemic. Future research may extend the scope of the proposed framework with additional constructs. Literature on COVID-19 presenting its impacts on health and wellness tourism consumption is at a development stage. Although our research provides significant support to develop the literature on the role of COVID-19 in the promotion of health and wellness tourism, we encourage future research to consider other literature on COVID-19, health and wellness tourism, and place attachment, which have been published as of the date of this review, to build on our initial conceptual model in this emerging field. This review introduces the concept of built landscapes which is related to the holistic medicine approach involving exercises, medical treatments, service providers’ caring attitudes at hospitals, and ambient environmental settings to address health and wellness tourists’ preferences. However, cultural landscapes, such as museums, or places with architecture goods might also impact on visitors’ well-being (good moods and happiness). We encourage future research to analyze the impact of cultural landscapes on health and wellness tourism consumption.

Due to the language proficiency of the researchers of this review, relevant literature was searched in English databases. There might be relevant and important literature on therapeutic landscapes in non-English databases. Future research may attempt to find relevant literature from non-English databases and may interpret them into English to fertile the grounds of theory and practice discussed in this review.

## Conclusion

Health and well-being promotion are linked to the constellation of services available at therapeutic landscapes. The ongoing COVID-19 pandemic has influenced pre-defined health and wellness philosophies. A deeper understanding of people’s perceptions of their physical and psychological needs in times of crises and disasters is essential. This may help to advance recovery of health and wellness tourist destinations, promote place attachment, and encourage re-visitation. Health and wellness tourism service providers will likely face challenging times post the COVID-19 pandemic. There is a need to revitalize the under-performing elements of health and wellness tourism destinations during COVID-19 and have further crisis management and recovery strategies in place.

## Author Contributions

SM and HR: conceptualization, literature gathering, literature analysis, development, revisions, and proofreading of the manuscript. Both authors: contributed to the article and approved the submitted version.

## Conflict of Interest

The authors declare that the research was conducted in the absence of any commercial or financial relationships that could be construed as a potential conflict of interest.
