# Supplementary material for: Health, Wellness, and Place Attachment During and Post Health Pandemics
Source: Front Psychol. 2020 Nov 26;11:573220. doi: 10.3389/fpsyg.2020.573220 (PMC7726477; doi:10.3389/fpsyg.2020.573220)
Supplement: Supplementary file 1 [file Table_1.docx]

Appendix 1

Sample spreadsheet entry

1

| Title: Natural places: perceptions of wildness and attachment to local greenspace |
| --- |
| Year of publication: 2019 |
| Type of literature: Journal article |
| Journal name: Journal of Environmental Psychology |
| Name of authors: Kathryn Colley & Tony Craig |
| Peer-reviewed: Yes |
| Extracted findings:   - Perceived naturalness impact affective attachment of everyday green spaces. - The design of natural landscapes of perceived places develops travelers’ positive attachment with the places. - The relationships between perceived landscapes, sense of place, and emotional attachment to place may offer valuable contribution to individuals’ health, well-being, and policymaking. |

2

| Title: Samadhi spa & wellness retreat: An Australian case study |
| --- |
| Year of publication: 2014 |
| Type of literature: Book chapter |
| Book title: Service Management in Health & Wellness Services |
| Name of authors: Haywantee Ramkissoon |
| Peer-reviewed: Yes |
| Extracted findings:   - Samadhi spa and wellness retreat offers holistic wellness treatments to nurture mind, body, heart, and soul. - Samadhi provides lifestyle treatments with health and happiness. - Samadhi meets expectations of visitors with high quality services, care, respect, and privacy. - Staff ranging from nutritionists, naturopaths, and sports physiologists, to name a few, is available at Samadhi to ensure visitors’ health and well-being. - Samadhi offers genuine care and kindness to satisfy visitors. - Samadhi offers spa, wellness, therapy, and spirituality with natural and built attractions. |

3

| Title: The antecedents of place attachment in the context of an Australian national park |
| --- |
| Year of publication: 2019 |
| Type of literature: Journal article |
| Journal name: Journal of Environmental Psychology |
| Name of authors: Carena J. van Riper, Jee In Yoon, Gerard T. Kyle, Kenneth E. Wallen, Adam C. Landon, Christopher Raymond |
| Peer-reviewed: Yes |
| Extracted findings:   - Place-based” motivations are the perceived benefits that emerge from opportunities afforded by an environment. - People with predominant nature-based worldviews are more likely to experience the benefits of nature, both psychological and physiological - Motivations facilitate the formation of attachment to a large natural environment. - The psychological, social, and physiological outcomes (i.e., “motivations”) of recreational experiences have been positioned as causal antecedents to place attachment. - The motivation-attachment relationship has drawn on expectancy-valence theory to operationalize people's experience preferences. - Settings vary in the opportunities they afford recreationists given heterogeneity in physical attributes, human histories, and regulatory frameworks that govern human-environment interactions. - Environmental worldviews underpin and help to explain the linkage between motivation and place attachment |

4

| Title: Place attachment and identification as predictors of expected landscape restorativeness |
| --- |
| Year of publication: 2019 |
| Type of literature: Journal article |
| Journal name: Journal of Environmental Psychology |
| Name of authors: Laura Menatti, Mikel Subiza-Perez, Arturo villalpando-Flores, Laura Vozmediano, Cesar San Juan |
| Peer-reviewed: Yes |
| Extracted findings:   - Person-place bonding affects preferences and the perception of landscape restorative properties - Place attachment consistently and positively predicted the assessment of the restorative properties of landscapes - Landscape preferences are overall judgements of attractiveness, aesthetics or scenic quality. - Landscape preferences are more likely in environments that offer prospects (or clear vantage points), and refuges (or hiding places), both of which allow human beings to be protected and survive - Place attachment is an emotional bond that people establish with significant places; that is, places they visit or use regularly and that make them feel at ease. - The perception of a place's restorativeness may be significantly influenced by an affective bond towards it. |

5

| Title: Pandemics, tourism and global change: a rapid assessment of COVID-19 |
| --- |
| Year of publication: 2020 |
| Type of literature: Journal article |
| Journal name: Journal of sustainable tourism |
| Name of authors: Stefan Gossling, Daniel Scott, C.Michael Hall |
| Peer-reviewed: Yes |
| Extracted findings:   - With the magnitude of the COVID-19 pandemic, there is an urgent need to reconsider the global tourism system for health and well-being. - The relationships between pandemics and travel are central to understanding health risks across global destinations promising health and well-being - Disease outbreaks such as SARS, Ebola, Marburg, hantavirus, Zika and avian influenza are all outcomes of anthropogenic impacts on ecosystems and biodiversity - One of the central realizations of research on pandemics is that travel is absolutely central to epidemiology and disease surveillance - travel and tourism is both a contributor to disease spread questioning travelers’ health and well-being |

6

| Title: Building attachments to places of settlement: A holistic approach to refugee wellbeing in Nelson, Aotearoa New Zealand |
| --- |
| Year of publication: 2019 |
| Type of literature: Journal article |
| Journal name: Journal of Environmental Psychology |
| Name of authors: Amber Kale |
| Peer-reviewed: Yes |
| Extracted findings:   - place-attachment is linked to wellbeing - Everyday multisensory environments impact feelings of safety and happiness of people - Built environments and natural environments allow diverse individuals encounter one another, have fun, and broaden their sensory palate. - Sensory perception develops affective personal and collective relationships with place |

7

| Title: Beyond nature: the roles of visual appeal and individual differences in perceived restorative potential |
| --- |
| Year of publication: 2019 |
| Type of literature: Journal article |
| Journal name: Journal of Environmental Psychology |
| Name of authors: Elyssa Twedt, Reuben M. Rainey, Dennis R. Proffitt |
| Peer-reviewed: Yes |
| Extracted findings:   - Built spaces may promote the well-being of people - Built spaces with restorative environment attract people seeking health and well-being. - However, natural spaces seem to be so universally positive - There is need to determine whether we can integrate natural attributes into built environments, taking into account limitations of space and cost across the world. - Person-environment interactions fuel perceived restoration process, provide a more flexible framework for designing restorative spaces, and contribute to a deeper understanding of the psychological impact of restorative or non-restorative environments under changing environmental and person circumstances. |

8

| Title: Therapeutic servicescapes: Restorative and relational resources in service settings |
| --- |
| Year of publication: 2020 |
| Type of literature: Journal article |
| Journal name: Journal of Retailing and Consumer Services |
| Name of authors: Mark S. Rosenbaum, Margareta Friman, German Contreras Ramirez, Tobias Otterbring |
| Peer-reviewed: Yes |
| Extracted findings:   - Therapeutic servicescapes offer consumers maintain bonds with or attachments to organizations providing health services - Patronage offers to consumers allow a type of catharsis, albeit temporarily, through restorative environmental conditions and meaningful relational interactions - The confluence of physical and social conditions occurs not only in ‘green’ or ‘blue’ spaces but also in commercial settings such as a grocery store, thus suggesting that servicescapes can have the same restoration potential as natural settings. - Most built and virtual commercial organizations are unable to create environments that simultaneously facilitate human restoration and offer consumers opportunities for rich social interaction with other people. - Need to build service settings to promote human restoration. |

9

| Title: Nature contact, nature connectedness and associations with health, wellbeing and pro-environmental behaviours |
| --- |
| Year of publication: 2020 |
| Type of literature: Journal article |
| Journal name: Journal of Environmental Psychology |
| Name of authors: Leanne Martin, Mathew P. White, Anne Hunt, Miles Richardson, Sabine Pahl, Jim Burt |
| Peer-reviewed: Yes |
| Extracted findings:   - Human health and wellbeing are linked to natural ecosystems - Maintaining contact with nature is positively related to an individual's own health and wellbeing, as well as their propensity to act in ways that protect the health of the planet. - Psychological connectedness with nature moderates the associations of human health and well-being. Nature contact may be more effective if accompanied by a positive disposition towards the environment. - Natural and built attractions are positively associated with people’s place connectedness. |

10

| Title: Place and behavior: the role of accessibility |
| --- |
| Year of publication: 2019 |
| Type of literature: Journal article |
| Journal name: Journal of Environmental Psychology |
| Name of authors: Dylan Bugden , Richard Stedman |
| Peer-reviewed: Yes |
| Extracted findings:   - Social context influences the relationship between sense of place and place-related behaviors - There is a significant relationship between sense of place and human behavior. - The sense of place-behavior relationship is automatic, inevitable, and robust. - Sense of place is often temporarily, rather than chronically, accessible in memory, i.e. the content, intensity, and availability of one's sense of place varies predictably according to physical and social contextual factors. - Mental representations, memory, and associative networks are central to the broader tradition of accessibility in social psychology. |

11

| Title: Ornamental indoor plants in hospital rooms enhanced health outcomes of patients recovering from surgery |
| --- |
| Year of publication: 2009 |
| Type of literature: Journal article |
| Journal name: The Journal of Alternative and Complementary Medicine |
| Name of authors: Seong-Hyun Park, Richard H. Mattson |
| Peer-reviewed: Yes |
| Extracted findings:   - Ambient environment, such as indoor plants, architect, had a positive influence during the recovery period of patients/visitors linking directly to health and well-being. - Patients/visitors exposed to plants during recovery had significantly enhanced physiologic responses evidenced by lower systolic blood pressure, lower ratings of pain, anxiety, and fatigue, and more positive feelings and higher satisfaction. - Patients/visitors exposed to ambient environment show better rating on health and well-being scale as compared to those exposed to non-ambient environment. - Built aesthetics positively influence peoples’ psychological filters and behaviors. |

12

| Title: Conceptualizing adventurous nature sport: A positive psychology perspective |
| --- |
| Year of publication: 2018 |
| Type of literature: Journal article |
| Journal name: Annals of Leisure Research |
| Name of authors: Susan Houge Mackenzie, Eric Brymer |
| Peer-reviewed: Yes |
| Extracted findings:   - Risk-focused approaches are narrow and do not account for the full range of motivations and outcomes associated with outdoor activities for health and well-being. - Positive psychology principles can be applied to conceptualize activities in natural and built environments as health and well-being activities - Adventurous natural locations could be considered in preventative health approaches as vehicles for wellness promotion. - Adventurous natural activities align with the movement toward ‘green prescriptions’ for health and may facilitate targeted well-being interventions for diverse populations. |

13

| Title: Indoor nature interventions for health and wellbeing of older adults in residential settings: A systematic review |
| --- |
| Year of publication: 2019 |
| Type of literature: Journal article |
| Journal name: The Gerontologist |
| Name of authors: Alison Bethel, Lewis R Elliott, Mathew P White, Nicola L Yeo, Ruth Garside, Sarah G Dean |
| Peer-reviewed: Yes |
| Extracted findings:   - Human-nature interaction promises mental health, social, and psychological wellbeing outcomes. - A combination of natural and built attractions inside the treatment facilities, such as indoor gardening programs, has greater potential to provide benefits than more passive exposures to aesthetic attractions. - Social interaction at healing places motivates people and promotes health and well-being. |

14

| Title: Psychological responses to natural patterns in architecture |
| --- |
| Year of publication: 2019 |
| Type of literature: Journal article |
| Journal name: Journal of Environmental Psychology |
| Name of authors: Alexander Coburn, Omid Kardan, Hiroki Kotabe, Jason Steinberg, Michael C. Hout, Arryn Robbins, Justin MacDonald, Gregor Hayn-Leichsenring, Marc G. Berman |
| Peer-reviewed: Yes |
| Extracted findings:   - The design of physical surroundings – including landscapes and buildings – can have a meaningful impact on psychological states and wellbeing. - The sensory qualities of natural environments have been found to improve mood, attention, and cognitive functioning. - Nature-like design features can be found in certain built environments that exhibit visual patterns inspired by biological systems. - Exposure to nature include improved mood, reduced stress, improved concentration and working memory performance, higher self-esteem, increased feelings of energy and vitality, and overall self-perceived health. Views of nature have also been shown to reduce criminal behavior and improve recovery from surgery. - Naturalistic forms and patterns have long served as a fruitful source of inspiration for architects and builders around the world. |

15

| Title: Authenticity: The link between destination image and place attachment |
| --- |
| Year of publication: 2016 |
| Type of literature: Journal article |
| Journal name: Journal of Hospitality Marketing & Management |
| Name of authors: Yangyang Jiang, Haywantee Ramkissoon, Felix T. Mavondo, Shanfei Feng |
| Peer-reviewed: Yes |
| Extracted findings:   - A significant relationship exists between destination image, existential authenticity and different dimensions of place attachment. - Visitors’ perceptions of authentic places develop destination image and place attachment. - Perceived authenticity of places offers good fit between destination image and place attachment, and visitors’ behaviors. |

16

| Title: Biophillic Design: How to enhance physical and psychological health and wellbeing in our built environments |
| --- |
| Year of publication: 2020 |
| Type of literature: Journal article |
| Journal name: Visions for Sustainability |
| Name of authors: Bettina Bolten, Giuseppe Barbiero |
| Peer-reviewed: Yes |
| Extracted findings:   - Biophilic Design is to design artificial environments that have a positive effect on individual health and well-being. - It is important to reconnect human beings with Nature for their overall health and well-being. - Biophilic Design touches on very deep parts of the human psyche, which are linked to the need, felt by many people, to - rediscover an affinity with Nature and feel at one with it again. - The association of built and natural environments allow people to recover their physical and mental more quickly and efficiently. |

17

| Title: Crisis management in tourism |
| --- |
| Year of publication: 2006 |
| Type of literature: Book chapter |
| Book title: Tourism Management Dynamics-Trends, management and tools |
| Name of authors: Tony S.M.Tse |
| Peer-reviewed: Yes |
| Extracted findings:   - The effective management of crises is a strategic necessity to reduce perceived risk of any tourism destination. - Crisis management helps to minimize and manage the impacts of crisis on visitors’ physical and mental health. - Effective communication on crisis management may help to develop positive image of the destinations. - Reduced perceived risk of destination crisis attracts tourists to destinations. |

18

| Title: Natural soundscapes in nature-based tourism: leisure participation and perceived constraints |
| --- |
| Year of publication: 2018 |
| Type of literature: Journal article |
| Journal name: Current Issues in Tourism |
| Name of authors: Jinde Jiang, Jie Zhang, Chunhui Zheng, Honglei Zhang, Junyi Zhang |
| Peer-reviewed: Yes |
| Extracted findings:   - Natural soundscapes is an attraction of nature-based tourism. - Natural soundscapes can improve the tourist experience. - Natural soundscapes positively influence tourists’ mental and physical health. - The perceived goodness of natural soundscapes promotes tourists revisits or loyalty behaviors towards the visited places. - The ‘lack of information, expectations and attraction’ might be the greatest constraints in promoting visitors’ positive attitudes. |

19

| Title: Psychological benefits of a biodiversity-focussed outdoor learning program for primary school children |
| --- |
| Year of publication: 2019 |
| Type of literature: Journal article |
| Journal name: Journal of Environmental Psychology |
| Name of authors: Deborah J. Harvey, Louise N. Montgomery, Hannah Harvey, Felix Hall, Alan C. Gange, Dawn Watling |
| Peer-reviewed: Yes |
| Extracted findings:   - Nature programs delivered in the built environment improve human beings wellbeing and mood, and enhance their connection to nature. - Exposure to nature ensures greater improvement in individuals’ health and wellbeing as compared to others not exposed to natural attractions in natural and built environments. - Exposure to nature may help individuals’ adopt a healthy lifestyle for a sustainable future. |

20

| Title: Neighbourhood amenities and health examining the significance of a local park |
| --- |
| Year of publication: 2013 |
| Type of literature: Journal article |
| Journal name: Social Science & Medicine |
| Name of authors: Jocelyn Plane, Fran Klodawsky |
| Peer-reviewed: Yes |
| Extracted findings:   - Social events and the ability to interact with others at the park promote people’s health and well-being. - Beautification certainly has value, community events are at least equally important to establish a good fit between visitors health and place attachment for overall health and well-being. - Places can be therapeutic and un-therapeutic depending on a number of interconnected place-specific and personal factors, such as risk of theft, residents’ drinking alcohol at parks. - Place in which one lives plays an important role in influencing their overall health and quality of life. - “Place”, however, refers not only to one’s physical dwelling, but also to public spaces such as the neighbourhood and the decision making practices that occur at many different levels of government that serve to influence access to neighbourhood-based social and economic resources. |

21

| Title: ‘Airplanes are flying nursing homes’: geographies in the concepts and locales of gerontological nursing practice |
| --- |
| Year of publication: 2005 |
| Type of literature: Journal article |
| Journal name: Journal of Clinical Nursing |
| Name of authors: Gavin J. Andrews, Dave Holmes, Blake Poland, Pascale Lehoux, Karen-Lee Miller, Dorothy Pringle, Katherine S. McGilton |
| Peer-reviewed: Yes |
| Extracted findings:   - A complex inter-relationship of human practices and physical settings make places social, cultural and symbolic phenomena, as well as sites for personal attachment and meaning leading to health and well-being. - Physical settings for the care of older people become places with a multitude of qualities, experiences and attachments. - Attending to place and nursing practice is a comprehensive approach to possibly improving , peoples’ collective experiences of nursing, health services, and feeling the perceived goodness of therapeutic relationships. |

22

| Title: (Re)thinking the dynamics between healthcare and place: therapeutic geographies in treatment and care practices |
| --- |
| Year of publication: 2004 |
| Type of literature: Journal article |
| Journal name: Area |
| Name of authors: Gavin J. Andrews |
| Peer-reviewed: Yes |
| Extracted findings:   - The mental imagery of places is an important therapeutic tool of complementary and alternative health practices. - Connections can be made to natural and idyllic places in order to calm and relax visitors. - Therapeutic images of places may be facilitated and assisted by the therapeutic environment of the clinic, its design and decoration. - Imagination of place also connects to improve the mind and body with the services of therapists and other service staff at therapeutic centers. - Therapeutic and landscape concepts are interlinked. |

23

| Title: Health tourism and hospitality: Spas, wellness and medical travel |
| --- |
| Year of publication: 2014 |
| Type of literature: Book |
| Publisher: Routledge, NY |
| Name of authors: **Melanie Smith**, László Puczkó |
| Reviewed by Publisher: Yes |
| Extracted findings:   - Health activities away from home, such as health tourism, improve people’s health and well-being. - Staying at leisure settings with exposure to sun and fun activities, sauna, massage, spa, and other health activities promote visitors’ health and well-being. - Medical treatments with alternative health practices at health places offering fun and adventure activities make tourists feel relaxed, improve mind, body, and soul, and perform better in daily activities. - Health places have therapeutic impacts on the body, mind, and spirit of visitors. - Individuals’ lifestyle and self-responsibility for health are paramount in the quest for a better quality of life. |

24

| Title: Simulated natural environments bolster the effectiveness of a mindfulness programme: A comparison on with a relaxation-based intervention |
| --- |
| Year of publication: 2020 |
| Type of literature: Journal article |
| Journal name: Journal of Environmental Psychology |
| Name of authors: Eun Yeong Choe, Anna Jorgensen, David Sheffield |
| Peer-reviewed: Yes |
| Extracted findings:   - The effect of the combination of the mindfulness programme and natural environments is greater than the effect of either the mindfulness programme in the non-natural environments or the relaxation group in natural environments. - People in the natural environments feel greater nature connectedness and satisfaction, lower negative feelings and reduced depression and stress compared with those in the non-natural environments. - Natural environment with a high degree of openness and accessibility are perceived as more restorative than one that was low in openness and accessibility. - Restorative effects of specific attributes of the natural environment would help to inform the design of well-being interventions and policies to improve public health and wellbeing |

25

| Title: Health and Wellness Tourism |
| --- |
| Year of publication: 2008 |
| Type of literature: Book |
| Publisher: Elsevier, Amsterdam |
| Name of authors: **Melanie Smith**, László Puczkó |
| Reviewed by Publisher: Yes |
| Extracted findings:   - Health and wellness tourism promotes tourists’ health and well-being. - Wellness treatments in natural and built environments establish biological connections of mind, body, and soul. - Health and wellness treatments in ambient environments fuels mental and physical health. - Visits to natural landscapes ensures good mood and less stress. |

26

| Title: Assessing the restorative potential of contemporary urban environment(s): beyond the nature versus urban dichotomy |
| --- |
| Year of publication: 2008 |
| Type of literature: Journal article |
| Journal name: Landscape and Urban Planning |
| Name of authors: Dmitri Karmanov, Ronald Hamel |
| Peer-reviewed: Yes |
| Extracted findings:   - Experiencing or simply viewing nature reduces stress and improves well-being. - Improved negative mood states, improved cognitive functioning, physiological signs of stress reduction, e.g. lower heart rate and muscle tension are some of the reported restorative effects following exposure to natural environments. - A strong affiliation with nature is considered to be of evolutionary origin in humans and it is manifested in a human being’s innate preferences for natural settings containing cues for water, food, and shelter. - Built settings, such as window views of green vegetation or water, rather than of other buildings or a brick wall, are found to be associated with improved attention capacity in adults. - Patients in hospital rooms with natural views requested less pain medication and recuperated faster following surgery than did patients whose room windows faced either other buildings or a brick wall. - The restorative potential of natural environments is well-established. |

27

| Title: Inpatient versus outpatient cervical priming for induction of labour: Therapeutic landscapes and women’s preferences |
| --- |
| Year of publication: 2011 |
| Type of literature: Journal article |
| Journal name: Health & Place |
| Name of authors: Candice Oster, Pamela L. Adelson, Chris Wilkinson, Deborah Turnbull |
| Peer-reviewed: Yes |
| Extracted findings:   - Therapeutic landscape experience offers the ‘‘positive physiological and psychological outcome deriving from a person’s imbrication within a particular socio-natural-material setting’’ in the home and hospital settings. - The home and hospital landscapes affect a sense of wellbeing through a network of factors. - Home and the hospital networks offer therapeutic benefits to people by providing them with a sense of wellbeing in different ways. - Places are therapeutic when people experience a healthy place-identity. - An understanding of physically comfortable and familiar places promotes mental and physical health as compared to the harsh, sterile and unfamiliar environments, such as the conventional hospitals. |

28

| Title: Immediate psychological responses and associated factors during the initial stage of the 2019 coronavirus disease (COVID-19) epidemic among the general population in China |
| --- |
| Year of publication: 2020 |
| Type of literature: Journal article |
| Journal name: International Journal of Environmental Research and Public Health |
| Name of authors: Cuiyan Wang, Riyu Pan, Xiaoyang Wan, Yilin Tan, Linkang Xu, Cyrus S. Ho, Roger C. Ho |
| Peer-reviewed: Yes |
| Extracted findings:   - Disease outbreaks directly impact people’s mental and physical health. - Destinations with rising cases of COVID-19 exert negative psychological impact on the residents and visitors. - People experience moderate-to-severe anxiety due to the perceived risk of COVID-19. - Residents and visitors of destinations hit by COVID-19 report higher levels of stress, anxiety, and depression. - Travel to destinations with COVID-19 is not preferred by the people in the ongoing context of pandemic. - Accurate health information and certain precautionary measures may help in lower psychological impact of the outbreak and lower levels of stress, anxiety, and depression. - Need to revitalize pandemic management efforts for improved psychological interventions to improve mental health and psychological resilience during the COVID-19 epidemic. |

29

| Title: Perception and evaluation of water in landscape: use of photo-projective method to compare child and adult residents’ perceptions of a Japanese river environment |
| --- |
| Year of publication: 2002 |
| Type of literature: Journal article |
| Journal name: Landscape and Urban Planning |
| Name of authors: Sampei Yamashita |
| Peer-reviewed: Yes |
| Extracted findings:   - Water in the landscape strongly attracts the attention of visitors and impacts their perceptions of the landscape. - Visual qualities of water impact tourists’ health and well-being and hold importance to be considered when planning and managing the landscape. - In perceptual psychological approach, water flow adds to scenic quality of landscapes. - Natural landscapes with water attractions hold attention of visitors of all age. |

30

| Title: Spaces of sobriety/sites of power: examining social model alcohol recovery programs as therapeutic landscapes |
| --- |
| Year of publication: 2006 |
| Type of literature: Journal article |
| Journal name: Social Science & Medicine |
| Name of authors: Robert Wiltona, Geoffrey DeVerteuil |
| Peer-reviewed: Yes |
| Extracted findings:   - Landscape of alcohol treatment and recovery is complex and multifaceted. - Good quality service treatments at landscapes of alcohol treatment and recovery may ensure visitors’ health and well-being. - The social relationship between staff and visitors is important to determine the mental health of the visitors. - The recovery landscape constituted through the concentration of professionals in recovery helps visitors quickly recover from alcohol addiction. |

31

| Title: Cultivating health: therapeutic landscapes and older people in northern England |
| --- |
| Year of publication: 2004 |
| Type of literature: Journal article |
| Journal name: Social Science & Medicine |
| Name of authors: Christine Milligan, Anthony Gatrell, Amanda Bingley |
| Peer-reviewed: Yes |
| Extracted findings:   - Therapeutic landscapes with gardens and gardening activity may contribute to the health and mental well-being of people. - Natural and built landscapes are very important for all age cohorts, especially aging population. - Natural landscape contributes positively, in both active and passive ways, to people’s mental well-being. - Therapeutic landscape gardens have restorative properties which influence visitors’ mental and physical health. - Visitors feel relaxed and may become emotionally connected to the landscapes. - Communal gardening activities for people can meet the needs of gardeners with a significant range of abilities and personal expectations. |

32

| Title: Buxton and the Peak District: Attracting visitors to the water festivals |
| --- |
| Year of publication: 2019 |
| Type of literature: Book chapter |
| Book title: Strategic Perspectives in Destination Marketing |
| Name of authors: Jessica Maxfield, Peter Wiltshier |
| Peer-reviewed: Yes |
| Extracted findings:   - Landscapes with natural attractions, such as water flow and sound, attract visitors. - Water flow in the natural environment positively influences the anatomical connection of mind, body, and spirit. - Natural landscapes with water attractions are considered important by wellness-seeking individuals. |

33

| Title: The geography of tourism and recreation, environment, place, and space |
| --- |
| Year of publication: 2002 |
| Type of literature: Book |
| Publisher: Routledge, NY |
| Name of authors: [C. Michael Hall](https://www.google.com.pk/search?tbo=p&tbm=bks&q=inauthor:%22C.+Michael+Hall%22), [Stephen J. Page](https://www.google.com.pk/search?tbo=p&tbm=bks&q=inauthor:%22Stephen+J.+Page%22) |
| Reviewed by Publisher: Yes |
| Extracted findings:   - Sustainable places with natural and built environments positively influence tourists` psychological filters. - Tourists` health and well-being notions are compromised due to environmental pollution, vehicles emitting pollution, and unhygienic surroundings. - Tourists` well-being depends on the quality of natural environment. - Recreational facilities with social gathering exert positive impacts on tourists` everyday behaviors. |

34

| Title: Blue space: the importance of water for preference, affect, and restorativeness ratings of natural and built scenes |
| --- |
| Year of publication: 2010 |
| Type of literature: Journal article |
| Journal name: Journal of Environmental Psychology |
| Name of authors: Mathew White, Amanda Smith, Kelly Humphryes, Sabine Pahl, Deborah Snelling, Michael Depledge |
| Peer-reviewed: Yes |
| Extracted findings:   - Relationships between aquatic environment and blue Space is evident in improving people`s health and well-being. - Natural and built settings with water attractions are associated with higher preferences and more positive subjective reactions of visitors of landscapes. - Urban planners, employers, mental and physical health professionals and educationalists may, for instance, need to consider the value of adding aquatic features to built environments, or arranging visits to natural aquatic environments, to promote the mental well-being of citizens, employees, patients and pupils. |

35

| Title: Perceptions of urban stream corridors within the greenway system of Sapporo, Japan |
| --- |
| Year of publication: 2004 |
| Type of literature: Journal article |
| Journal name: Landscape and Urban Planning |
| Name of authors: Shoichiro Asakawa, Keisuke Yoshida, Kazuo Yabe |
| Peer-reviewed: Yes |
| Extracted findings:   - Stream corridors into greenway improves people`s mental health. - Places with stream corridors into greenways ensure mental and physical health of nearby residents and visitors. - People`s perceptions toward the stream corridors are recreational use, participation, nature and scenery, sanitary maintenance, and water safety. - Topography (alluvium fan and lowland), distance from hills and mountains, as well as surrounding land uses are important contexts in the rating of nature and scenery of landscape for people`s health and well-being. |

36

| Title: Therapeutic experiences of community gardens: Putting flow in its place |
| --- |
| Year of publication: 2014 |
| Type of literature: Journal article |
| Journal name: Health & Place |
| Name of authors: Hannah Pitt |
| Peer-reviewed: Yes |
| Extracted findings:   - Community gardens are a combination of natural and built environment - Community gardens have much in common with other places celebrated as therapeutic landscapes. - Community gardens allow physical and mental distance from stress. - Gardening is therapeutic not through passive presence in place but through moving in ways conducive to intensely focused moments of absorption in skilled rhythmic activities. - The relationship between individuals` wellbeing and place is not the sole determinant of his/her condition. - Health and wellness service providers add to therapeutic place experiences and the extent to which a restorative moment brings enduring wellbeing benefits. |

37

| Title: Objective versus subjective assessments of environmental quality of standing and running waters in a large city |
| --- |
| Year of publication: 2008 |
| Type of literature: Journal article |
| Journal name: Landscape and Urban Planning |
| Name of authors: Astrid Steinwender, Claudia Gundacker, Karl J. Wittmann |
| Peer-reviewed: Yes |
| Extracted findings:   - Age and mood of visitors determine the perceptual criteria for further assessment of the quality of landscapes. - Natural environment, such as cloudiness, wind, and air temperature, of landscapes exerts positive impacts on the health and well-being of visitors. - Perceptions of naturalness and aesthetic quality of waters were more strongly influenced by subjective components than perceptions of smell and visual turbidity. - Water management projects improve public acceptance by complementary measures such as community participation, public presentation and/or opinion polls before project implementation. |

38

| Title: Healing places |
| --- |
| Year of publication: 2003 |
| Type of literature: Book |
| Publisher: Rowman and Littlefield |
| Name of authors: Wilbert M Gesler |
| Reviewed by Publisher: Yes |
| Extracted findings:   - Environmental stimuli impact people`s perceptions. - Natural, built, social, and symbolic environments are noted as healing environments which directly impact people`s health and well-being. - A network of service providers, such as health professionals of the eastern and the western medical treatments, at therapeutic landscapes determine mental and physical health of visitors. - A good fit between healing places and exposure to spend time with other people, i.e., social interaction, fuels perceived goodness of the restorative capacity of the therapeutic landscapes. |

39

| Title: Blue care: a systematic review of blue space interventions for health and wellbeing |
| --- |
| Year of publication: 2018 |
| Type of literature: Journal article |
| Journal name: Health Promotion International |
| Name of authors: Easkey Britton, Gesche Kindermann, Christine Domegan, Caitriona Carlin |
| Peer-reviewed: Yes |
| Extracted findings:   - Activities in blue space, rather than particular qualities of blue space, might contribute to rehabilitation and health promotion. - Activities in blue spaces have significant positive effects for health, especially psycho-social wellbeing benefits, with relatively few findings for physical health. - Blue care has the potential to improve mental health of all age cohorts. |

40

| Title: Nature-based therapeutic interventions |
| --- |
| Year of publication: 2010 |
| Type of literature: Book chapter |
| Book title: Forest, trees and human health |
| - Name of authors: Ulrika K. Stigsdotter, Anna Maria Palsdottir, Ambra Burls, Alessandra Chermaz, Francesco Ferrini, Patrik Grahn |
| Peer-reviewed: Yes |
| Extracted findings:   - Nature-based settings as an important asset for improvement and promotion of health - The concepts of healthy nature-based settings and accompanying treatment programs have been referred to health treatments, alternative treatments, natural therapies, etc. - The quality therapy program and the health design of the nature-based setting may ensure people’s health and well-being. |

41

| Title: People and nature: Toward an ecological model of health promotion |
| --- |
| Year of publication: 2010 |
| Type of literature: Invited Essay |
| Journal name: Leisure Sciences |
| Name of authors: Daniel L. Dustin, Kelly S. Bricker, Keri A. Schwab |
| Peer-reviewed: Yes |
| Extracted findings:   - People’s physiological, psychological, and spiritual health lies in the natural world. - The associations of park, recreation, tourism, and professionals’ skills may overcome many health-related problems. - Natural and built settings away from the urbanized way of life may promote people’s health and well-being. |

42

| Title: Pharmaceuticals and tourist spaces: Encountering the medicinal in Cozumel’s linguistic landscape |
| --- |
| Year of publication: 2017 |
| Type of literature: Journal article |
| Journal name: ACME: An International Journal of Critical Geographies |
| Name of authors: Leon Hoffman |
| Peer-reviewed: Yes |
| Extracted findings:   - Experience of therapeutic landscapes is complex to determine. - The pharmaceutical features of a tourism destination appear to be enmeshed within discourses that facilitate broader touristic landscape development for visitors’ health and well-being. - Service providers, such as insignia, give identity to pharmacies, and helps advertise the products available inside, appears to blend and engage with the surrounding tourism activity, promoting medicinal goods as a kind of souvenir alongside myriad gifts and trinkets symbolic and self-affirming of the tourist experience. - Medical becomes part of the tourist landscape, a consumer experience which presses to offer tourists that which might not be available at home and/or a memento of their trip abroad. |

43

| Title: Place-making, settlement and well-being: The therapeutic landscapes of recently arrived youth with refugee backgrounds |
| --- |
| Year of publication: 2010 |
| Type of literature: Journal article |
| Journal name: Health & Place |
| Name of authors: Robyn Sampson, Sandra M. Gifford |
| Peer-reviewed: Yes |
| Extracted findings:   - Places of opportunity, places of restoration, places of sociability and places of safety are particularly important to people on arrival and which together can be understood to constitute therapeutic landscapes through their contribution to experiences of restoration and renewal. - These four types of places promote people’s health-optimizing activities at therapeutic landscapes. - The restorative characteristics of place and place-making have the capacity to contribute to recovery and well-being. - People feel connected to therapeutic landscapes and spend time to experience the restorative environment. |

44

| Title: Stakeholders’ views of enclave tourism. A grounded theory approach |
| --- |
| Year of publication: 2013 |
| Type of literature: Journal article |
| Journal name: Journal of Hospitality & Tourism Research |
| Name of authors: Robin Nunkoo, Haywantee Ramkissoon |
| Peer-reviewed: Yes |
| Extracted findings:   - Enclave tourism has some benefits with respect to environmental management, local entrepreneurship, tourist’ health and well-being, and local empowerment, which could be the basis for sustainable tourism development. - Hotel resort enclaves invest a lot in environmentally friendly and sustainable practices to meet tourists’ needs of optimal health and well-being. - Stakeholders’ of hotel resort need to combine their service skills to offer appropriate health and well-being experiences to tourists. |

45

| Title: Encountering place: A psychoanalytic approach for understanding how therapeutic landscapes benefit health and wellbeing |
| --- |
| Year of publication: 2012 |
| Type of literature: Journal article |
| Journal name: Health & Place |
| Name of authors: Emma Rose |
| Peer-reviewed: Yes |
| Extracted findings:   - Individual can encounter landscape as an empathic mirror of feeling states and affects, enabling him or her to gain a broader appreciation of and competence with the emotional and cognitive conditions of subjective and inter-subjective well-being. - Therapeutic landscape, then, helps to establish the possibility for a pretend mode of viewing. - Actual viewing of landscapes is very important for the human health and well-being. - A combination of natural and built landscapes alongside service providers’ professional skills help to maintain people’s good health and re-visits. |

46

| Title: The landscape of caring for women: a narrative study of midwifery practice |
| --- |
| Year of publication: 2004 |
| Type of literature: Journal article |
| Journal name: Journal of Caring for Women |
| Name of authors: Holly Powell Kennedy, Maureen T. Shannon, Usa Chuahorm, M. Kathryn Kravetz |
| Peer-reviewed: Yes |
| Extracted findings:   - Health care system need to balance rising costs with an ever-increasing reliance on, and demand for, technological innovation. - Health service providers’ skills largely determine people’s health and well-being. - Health workers’ professional, a personal connection, partnership, and often friendly services add fuel to the quick recovery of patients or visitors. |

47

| Title: Religion/spirituality, therapeutic landscape and immigrant mental well-being amongst African immigrants to Canada |
| --- |
| Year of publication: 2016 |
| Type of literature: Journal article |
| Journal name: Mental Health, Religion & Culture |
| Name of authors: Boadi Agyekum & Bruce K. Newbold |
| Peer-reviewed: Yes |
| Extracted findings:   - Religious places hold importance as therapeutic landscapes in shaping people’s health and well-being. - Religious sites and activities impact members’ lives through multiple pathways, including physical, social, emotional, spiritual and mental well-being through everyday activities in these “healthy spaces”. - Religious places and practices are significant for health, mental wellness and general quality of life amongst immigrants and locals. - Religious involvement and perception of mental wellness are similar across different destinations. - Therapeutic landscape lens support the contention that religious sites are sources of healing and recovery. |

48

| Title: Spiritual retreat tourism in New Zealand |
| --- |
| Year of publication: 2013 |
| Type of literature: Journal article |
| Journal name: Tourism Recreation Research |
| Name of authors: Kate Bone |
| Peer-reviewed: Yes |
| Extracted findings:   - Spiritual retreats function as therapeutic landscapes to ensure people’s health and well-being. - Various aspects of spiritual retreats combine to create a holistic experience for tourists who desire going somewhere with a sense of community - Spiritual retreats offer visitors enjoying an escape from everyday life, consuming the aesthetically attractive and symbolically rich landscape of retreats, and being in a spiritually accepting environment. - The experiences of therapeutic places, spirituality and well-being for retreat tourists are some of the features alike wellness tourism to ensure health and well-being of tourists/visitors. |

49

| Title: Spiritual therapeutic landscapes and healing: A case study of St. Anne de Beaupre, Quebec, Canada |
| --- |
| Year of publication: 2010 |
| Type of literature: Journal article |
| Journal name: Social Science & Medicine |
| Name of authors: Allison Williams |
| Peer-reviewed: Yes |
| Extracted findings:   - The characteristics of traditional therapeutic landscapes are broadly categorized into the notions of treatment and/or healing. - Visiting the spiritual sites, such as shrines, conforms to the healing and recovery notions of therapeutic landscapes. - Pilgrims, even if only visiting for a short time, partake in numerous embodied rituals to ensure healing, health and wellbeing. - The experience of visiting religious site, via the embodied participation, provided emotional relief and spiritual replenishment. |

50

| Title: Surfing therapeutic landscapes: Exploring cyberpilgrimage |
| --- |
| Year of publication: 2013 |
| Type of literature: Journal article |
| Journal name: Culture and Religion: An Interdisciplinary Journal |
| Name of authors: Allison M. Williams |
| Peer-reviewed: Yes |
| Extracted findings:   - Built landscape environments even in virtual settings positively influence viewers’ psychological health. - Virtual therapeutic landscapes attempt to provide replacement to actual visits to remote places. - Without actual journey, the virtual pilgrim still feels as though they have had an accurate portrayal of the sites. - Virtual tours show improvement in viewers’ mental health that further support physical health. |

51

| Title: Theoretical injections: On the therapeutic aesthetics of medical spaces |
| --- |
| Year of publication: 2009 |
| Type of literature: Journal article |
| Journal name: Social Science & Medicine |
| Name of authors: Joshua D. Evans, Valorie A. Crooks, Paul T. Kingsbury |
| Peer-reviewed: Yes |
| Extracted findings:   - Environmental artwork in hospital waiting rooms positively influences patients’ physical and mental health. - Aesthetic feeling at therapeutic centers adds meanings to visitors’ quality of life. - waiting room is a complex ‘space of public display’ that through its design exposes some of our deepest assumptions, or perhaps more accurately our deepest illusions, about ourselves and our bodies. - The presence of environmental artwork in the hospital may be there as much for the benefit of health care providers as for the patients who gaze upon it. - Putting nature on display in the waiting room diverts occupants’ thoughts away from the Real while simultaneously reinforcing another gaze. - The role of the geographical gaze (i.e., waiting room occupants’ views upon the environmental artwork that displays images of other places) is to immerse people in feelings and sensations of calm, relaxation, escape, and even pleasure in a way that shifts attention away from the very real ‘goings on’ within the hospital at large. |

52

| Title: Therapeutic landscapes and postcolonial theory: A theoretical approach to medical tourism |
| --- |
| Year of publication: 2012 |
| Type of literature: Journal article |
| Journal name: Social Science & Medicine |
| Name of authors: Christine N. Buzinde, Careen Yarnal |
| Peer-reviewed: Yes |
| Extracted findings:   - Medical tourism destination and their construction of therapeutic landscapes are important to explore to appropriately understand tourists’ desires of optimal health and well-being. - Periphery/core relationships characterize medical tourism sites as trans-cultural spaces of care. - Drawing on scholarship on therapeutic landscapes and postcolonial theory, the micro and macro level issues can be examined pertaining to power, discourse and representation that are intricately linked to the construction and production of spaces of healing located in periphery nations. - Emphasis on macro level issues would facilitate examinations of the discursive construction of nations as curative landscapes, spaces of leisure and travel, and/or regions in which the medical discourse of modernity and scientific progress resonate. |

53

| Title: From therapeutic landscapes to healthy spaces, places and practices: A scoping review |
| --- |
| Year of publication: 2018 |
| Type of literature: Journal article |
| Journal name: Social Science & Medicine |
| Name of authors: Sarah L. Bella, Ronan Foley, Frank Houghton, Avril Maddrell, Allison M. Williams |
| Peer-reviewed: Yes |
| Extracted findings:   - Therapeutic landscapes are stretched around the core material, social, spiritual and symbolic dimensions. - The health and healing benefits of space and place are important for visitors’ health and well-being. - The role of green and blue spaces is important in the development of restorative capacities of therapeutic landscapes. - Health education and promotion initiatives, within which elements of outdoor exercise like yoga, or embodied mobilities like walking, are enacted in and through green space are vital antecedents to visitors’ health and well-being. - Originally subsumed within green space, blue spaces have become sites of increased attention, with water at the centre of a range of outdoor spaces perceived to promote healthy living. - ‘Blue’ settings (islands, cities, rivers, coasts, beaches, lakes) and practices (swimming, promenading, retirement, walking) ensure visitors’ health and well-being, first and repeat visits, and place attachment. |

54

| Title: A therapeutic taskscape: Theorizing place-making, discipline and care at a camp for troubled youth |
| --- |
| Year of publication: 2009 |
| Type of literature: Journal article |
| Journal name: Health & Place |
| Name of authors: Cheryl Morse Dunkley |
| Peer-reviewed: Yes |
| Extracted findings:   - Camps are identified for youth as therapeutic landscapes. - Camps as landscapes designed to promote positive growth for young people within the spaces of nature. - Therapeutic camping is a subset of a group of practices variously referred to as wilderness therapy (WT), adventure therapy or ‘outdoor behavioral healthcare programs’. - Literature on wilderness therapy is concerned with measuring outcomes and behavioral change of WT participants. - WT programs for people with problem behaviors attempt to prompt behavioral change by placing them in an outdoor setting, engaging them in challenging activities, and processing or ‘debriefing’ the experience using some form of behavioral and/or cognitive therapy. |

55

| Title: Therapeutic landscape and longevity: Wellness tourism in Bama |
| --- |
| Year of publication: 2018 |
| Type of literature: Journal article |
| Journal name: Social Science & Medicine |
| Name of authors: Liyuan Huang, Honggang Xu |
| Peer-reviewed: Yes |
| Extracted findings:   - Longevity reflects the holistic concept that health and place operate together as an integrated system. - Natural environment, social interaction and symbolic landscape work together in the healing process of the tourists and adds meanings to longevity notion. - In a wellness tourism destination, tourists actively participate in the production of the therapeutic landscape and facilitate the healing process. - Tourism enhances the potential spiritual linkage between tourists and the destination for health and well-being. - Alternative Chinese traditional health practices have influenced health and wellness tourists’ preferences of optimal health and well-being. |

56

| Title: Medical geography: therapeutic places, spaces and networks |
| --- |
| Year of publication: 2005 |
| Type of literature: Journal article |
| Journal name: Progress in Human Geography |
| Name of authors: Fiona Smyth |
| Peer-reviewed: Yes |
| Extracted findings:   - The physical, social and symbolic landscapes of therapeutic environments (including places, spaces and networks) serve to regulate and normalize visitors’ behaviors. - The coherent coordination of therapeutic landscapes and networks significantly influence health-related behaviors of visitors. - A space can be exposed to people to give way to new opportunities to develop landscapes of resistance and activity that challenge exclusionary discourses about ethnicity, race, impairment and gender and discourses about particular conditions such as psychiatric disorders, HIV/AIDS and other sexually transmitted infections. |

57

| Title: Therapeutic landscapes and wellbeing in later life: Impacts of blue and green spaces for older adults |
| --- |
| Year of publication: 2015 |
| Type of literature: Journal article |
| Journal name: Health & Place |
| Name of authors: Jessica Finlay, Thea Franke, Heather McKay, Joanie Sims-Gould |
| Peer-reviewed: Yes |
| Extracted findings:   - The interaction of green and blue spaces is important in the promotions of people’s health and well-being. - People feel motivated to get out of the house to exercise, enjoy the fresh air, and develop social interaction with others. - Therapeutic landscapes improve mental wellbeing, feelings of renewal, rejuvenation, and restoration. - A combination of blue and green space help people to feel relaxed, contemplated, and connected spiritually to their loved ones. - These spaces enhanced social wellbeing by providing opportunities for social interaction and engagement, especially in green spaces. |

58

| Title: Selling the private asylum: therapeutic landscapes and the (re)valorization of confinement in the era of community care |
| --- |
| Year of publication: 2006 |
| Type of literature: Journal article |
| Journal name: Transactions of the Institute of British Geographers |
| Name of authors: Graham Moon, Robin Kearns, Alun Joseph |
| Peer-reviewed: Yes |
| Extracted findings:   - Psychiatric care settings in terms of their material and symbolic construction as therapeutic landscapes may help to improve people’s mental health. - Tourism attractions such as hot Springs in South Dakota or a spa town may ensure the anatomical connection of people’s mind, body, and soul. - There is a clear importance that can be attached to the promotion of active minds and active bodies within healthy settings composed of green and blue spaces. |

59

| Title: Therapeutic mobilities: walking and ‘steps’ to wellbeing and health |
| --- |
| Year of publication: 2013 |
| Type of literature: Journal article |
| Journal name: Health & Place |
| Name of authors: Anthony C. Gatrell |
| Peer-reviewed: Yes |
| Extracted findings:   - Mobilities can be therapeutic. - Places ‘enable’, but so does movement; not in the simple sense of ‘enabling’ transport from one place to another, but in the sense of bringing together place-specific ‘unique association of social, affective and material resources. - Walking in therapeutic landscapes improves physical fitness and mental health. - Therapeutic landscapes cement existing, or develops new, friendships and social interactions; alongside promoting health and wellbeing. - Mobility supports wellbeing, and wellbeing permits mobility. |

60

| Title: ‘No Ebola….still doomed’ – The Ebola-induced tourism crisis |
| --- |
| Year of publication: 2018 |
| Type of literature: Journal article |
| Journal name: Annals of Tourism Research |
| Name of authors: Marina Novellia, Liv Gussing Burgess, Adam Jones, Brent W. Ritchie |
| Peer-reviewed: Yes |
| Extracted findings:   - Heath related crises could influence tourist risk perception. - Heath related crises could discourage tourists’ from traveling to tourism destinations for health and well-being. - Crisis-hit destinations may face fall in their economic share from the tourism. - Crises exert long-lasting impacts on the reputation of destinations promoting health and well-being. |

61

| Title: Tourism and wellbeing |
| --- |
| Year of publication: 2017 |
| Type of literature: Journal article |
| Journal name: Annals of Tourism Research |
| Name of authors: Melanie Kay Smith, Anya Diekmann |
| Peer-reviewed: Yes |
| Extracted findings:   - Sun, sea, and sand notions are important and complex to determine in the relationship between tourism and tourists’ well-being. - People need a chance to relax and recuperate on holiday at green and blue landscapes and episodic happiness and hedonic pleasures are an integral part of this. - The combination of nature and tourism promises health and well-being of tourists. - Tourists prefer to visit green and blue landscapes to experience the restorative powers of natural enviromnet. |

62

| Title: Tourism and wellbeing |
| --- |
| Year of publication: 2017 |
| Type of literature: Journal article |
| Journal name: Annals of Tourism Research |
| Name of authors: Melanie Kay Smith, Anya Diekmann |
| Peer-reviewed: Yes |
| Extracted findings:   - Sun, sea, and sand notions are important and complex to determine in the relationship between tourism and tourists’ well-being. - People need a chance to relax and recuperate on holiday at green and blue landscapes and episodic happiness and hedonic pleasures are an integral part of this. - The combination of nature and tourism promises health and well-being of tourists. - Tourists prefer to visit green and blue landscapes to experience the restorative powers of natural environment. |

63

| Title: Seeking everyday wellbeing: The coast as a therapeutic landscape |
| --- |
| Year of publication: 2015 |
| Type of literature: Journal article |
| Journal name: Social Science & Medicine |
| Name of authors: Sarah L. Bell, Cassandra Phoenix, Rebecca Lovell, Benedict W. Wheeler |
| Peer-reviewed: Yes |
| Extracted findings:   - Green space impacts people`s well-being. - Coastal experience improves people`s everyday life. - People`s become emotionally attached to coastal lines. - There is need to nurture diversity along the coastline, catering for wide-ranging needs and interests to minimize sensations of crowding and maximize opportunities for individuals. |

66

| Title: Using geonarratives to explore the diverse temporalities and therapeutic landscapes: Perspectives from ‘green’ and ‘blue’ settings |
| --- |
| Year of publication: 2016 |
| Type of literature: Journal article |
| Journal name: Annals of the Association of American Geographers |
| Name of authors: Sarah L. Bell, Benedict W. Wheeler & Cassandra Phoenix |
| Peer-reviewed: Yes |
| Extracted findings:   - Interactions with local green and blue settings can fluctuate according to new shared needs, capabilities, and preferences as a result of these relationships. - Landscapes “where the physical and built environments, social conditions, and human perceptions combine to produce an atmosphere which is conducive to healing. - Places, such as sacred pilgrimage sites, holy wells, and springs improve people`s health and well-being. - Everyday settings contribute to ill health prevention as well as healing, exploring how dynamic material, social, cultural, and personal assemblages entwine to shape a sense of wellbeing at particular times and for certain individuals and groups. - “ordinary” everyday assemblages, deemed integral to the routine self-management of individual well-being over time, include, for example, community gardens, coastlines, woodland, and riverside environments. |

67

| Title: Pampering, well-being and women’s bodies in the therapeutic spaces of the spa |
| --- |
| Year of publication: 2013 |
| Type of literature: Journal article |
| Journal name: Social & Cultural Geography |
| Name of authors: Jo Little |
| Peer-reviewed: Yes |
| Extracted findings:   - Pampering provides an interesting example of the intersection between health and well-being and the appropriate body. - Pampering is often used as a rather ‘catch-all’ term for describing a range of practices in which individuals are indulged and through which their bodies are ‘treated’ using a variety of (often small scale) luxuries. - Such luxuries take the form of products (oils, lotions, towels) and environments (use of music, comfortable seating, attractive de´cor) - pampering is considered as a therapy of bodily maintenance and care, designed to enhance the physical and mental well-being of the individual, and act as a way of disciplining and controlling the body. - Modern day spa plays a valuable role in women’s ability to access pampering as an activity associated with both well-being and beauty. |

68

| Title: You have to make a new way of life’: women’s drug treatment programmes as therapeutic landscapes in Canada |
| --- |
| Year of publication: 2012 |
| Type of literature: Journal article |
| Journal name: Gender, Place and Culture |
| Name of authors: Love, M., Wilton, R., and DeVerteuil, G |
| Peer-reviewed: Yes |
| Extracted findings:   - Drug treatment and recovery at therapeutic centers are complex and multifaceted in nature. - Good quality service treatments at landscapes of drug treatment and recovery may ensure women’s health and well-being. - The social relationship between staff and visitors is important to determine the mental and physical health of the visitors. - The recovery landscape constituted through the concentration of professionals in recovery helps visitors quickly recover from drug addiction. |

69

| Title: The ‘world of yoga’: The production and reproduction of therapeutic landscapes |
| --- |
| Year of publication: 2007 |
| Type of literature: Journal article |
| Journal name: Social Science & Medicine |
| Name of authors: Hoyez, A.-C |
| Peer-reviewed: Yes |
| Extracted findings:   - Therapeutic landscapes with exercise opportunities involve material, social, spiritual and symbolic dimensions. - The health and healing benefits of space, place, and yoga are important for visitors’ health and well-being. - The role of green and blue spaces with yoga and other alternative health treatments is important in the development of restorative capacities of therapeutic landscapes. - Health education and promotion initiatives, within which elements of outdoor exercise like yoga, or embodied mobilities like walking, are enacted in and through green space are vital antecedents to visitors’ health and well-being. - Built and natural environments are important for first/repeat visitors’ attention and to promote their healthy living. - ‘Blue’ settings (islands, cities, rivers, coasts, beaches, lakes) and practices (swimming, promenading, retirement, walking) ensure visitors’ health and well-being, first and repeat visits, and place attachment. |

70

| Title: Therapeutic landscapes, Health, and Healing |
| --- |
| Year of publication: 2014 |
| Type of literature: Book chapter |
| Book title: The Wiley Blackwell Encyclopedia of Health, Illness, Behavior, and Society |
| Name of authors: Williams, A.M |
| Peer-reviewed: Yes |
| Extracted findings:   - Therapeutic landscapes with gardens, community centers, therapy centers for social activities may contribute to the health and mental well-being of people. - Natural and built landscapes positively influence visitors’ moods. - Natural landscape contributes positively, in both active and passive ways, to people’s mental well-being. - Therapeutic landscape gardens have restorative properties which influence visitors’ mental and physical health. - Visitors feel relaxed and may become emotionally connected to the landscapes. |

71

| Title: The impact of blue space on human health and well-being-Salutogenetic health effects of inland surface waters: A review |
| --- |
| Year of publication: 2011 |
| Type of literature: Journal article |
| Journal name: International Journal of Hygiene and Environmental Health |
| Name of authors: Völker, S., and Kistemann, T. |
| Peer-reviewed: Yes |
| Extracted findings:   - Visitors’ attention and perceptions are strongly influenced water flow of landscape. - Visual qualities of water impact people’s health and well-being. - In perceptual psychological approach, water flow adds to scenic quality of landscapes. - Natural landscapes with water attractions hold attention of visitors of all age. |

72

| Title: Tourism and health: using positive psychology principles to maximize participants’ wellbeing outcomes – a design concept for charity challenge tourism |
| --- |
| Year of publication: 2015 |
| Type of literature: Journal article |
| Journal name: Journal of Sustainable Tourism |
| Name of authors: Coghlan, A. |
| Peer-reviewed: Yes |
| Extracted findings:   - Tourism destinations with natural and built attractions positively influence tourists` physical and mental health. - Tourists` health and well-being notions are ensures with health and wellness activities. - Tourists` satisfaction is susceptible to the availability of charity giving activities that develop inner satisfaction and peace. - Social interactions is developed in activities, such as fund raising for charity, at tourism destinations which help to find inner peace, satisfaction, and happiness that may positive impact on tourists` everyday behaviors. |

73

| Title: Performing health in place: The holy well as a therapeutic assemblage |
| --- |
| Year of publication: 2010 |
| Type of literature: Journal article |
| Journal name: Health & Place |
| Name of authors: Foley, R. |
| Peer-reviewed: Yes |
| Extracted findings:   - Therapeutic assemblages are landscapes “where the physical and built environments, social conditions, and human perceptions combine to produce an atmosphere which is conducive to healing. - Places, such as holy wells, and springs improve people`s health and well-being. - Everyday settings contribute to ill health prevention as well as healing to shape a sense of wellbeing. - Holy wells are an integral part of individual well-being over time due to its restorative properties. |

74

| Title: Healing in places of decline: (Re)imagining everyday landscapes in Hamilton, Ontario |
| --- |
| Year of publication: 2005 |
| Type of literature: Journal article |
| Journal name: Health & Place |
| Name of authors: Wakefield, S., and McMullan, C. |
| Peer-reviewed: Yes |
| Extracted findings:   - Perceived naturalness impact affective attachment of everyday green spaces. - Recreational facilities with social gathering exert positive impacts on tourists` everyday behaviors. - Health activities impact members’ lives through multiple pathways, including physical, social, emotional, spiritual and mental well-being through everyday activities in these “healthy spaces”. |

75

| Title: Seniors’ seasonal movement for health enhancement |
| --- |
| Year of publication: 2017 |
| Type of literature: Journal article |
| Journal name: The Service Industries Journal |
| Name of authors: Zhou, L., Yu, J., Wu, M.-Y., Wall, G., and Pearce, P. L. |
| Peer-reviewed: Yes |
| Extracted findings:   - Natural activities that align with the movement toward ‘green prescriptions’ for health and may facilitate targeted well-being interventions for seniors. - Exercises and movement opportunities are important to develop attractions of landscapes. - Movement and social activities at healing places may help seniors in the promotion of mind, body, and soul. |

76

| Title: Modelling spa-goers’ choice of therapeutic activities |
| --- |
| Year of publication: 2017 |
| Type of literature: Journal article |
| Journal name: Journal of Hospitality and Tourism Management |
| Name of authors: Charles Atanga Adongo, Francis E. Amuquandoh, Edem Kwesi Amenumey |
| Peer-reviewed: Yes |
| Extracted findings:   - Spa facilities at therapeutic landscapes offer holistic wellness treatments to nurture mind, body, heart, and soul. - Spa is considered as lifestyle treatments to improve health and happiness. - Spa service providers ensure visitors’ health and well-being. - Wellness, therapy, such as spa, offers spiritual well-being at natural and built attractions. |

77

| Title: Health, healing and recovery: Therapeutic landscapes and the everyday lives of breast cancer survivors |
| --- |
| Year of publication: 2008 |
| Type of literature: Journal article |
| Journal name: Social Science & Medicine |
| Name of authors: Jennifer English, Kathi Wilson, Sue Keller-Olaman |
| Peer-reviewed: Yes |
| Extracted findings:   - Therapeutic landscapes are extraordinary places of healing. - Therapeutic landscapes offer health and wellness treatments to visitors to improve their everyday activities. - Therapeutic landscapes offer restorative environments to critical disease patients, such as breast cancer survivors. |

78

| Title: Therapeutic landscapes and living with breast cancer: The lived experience of Thai women |
| --- |
| Year of publication: 2015 |
| Type of literature: Journal article |
| Journal name: Social Science & Medicine |
| Name of authors: Pranee Liamputtong, Dusanee Suwankhong |
| Peer-reviewed: Yes |
| Extracted findings:   - The role of everyday and extraordinary landscapes is important to note in shaping the healing experiences of women surviving breast cancer revealing the multiple healing landscapes women draw on for their recovery from breast cancer. - Therapeutic landscapes positively influence people’s emotions, suffering from critical diseases such as breast cancer, with strong cultural beliefs and practices. - Health care providers need to understand the emotional experiences of women with breast cancer and their particular cultural needs for emotional healing landscapes. - Health care providers need to respond to individual emotional experiences and needs for healing among women with breast cancer to ensure their repeat visits. |

79

| Title: Therapeutic mobilities |
| --- |
| Year of publication: 2019 |
| Type of literature: Journal article |
| Journal name: Mobilities |
| Name of authors: Heidi Kaspar, Margaret Walton-Roberts & Audrey Bochaton |
| Peer-reviewed: Yes |
| Extracted findings:   - Therapeutic mobilities include the holistic concepts of transnationally circulating patients, health professionals and pharmaceuticals for people’s health and well-being. - The integrated coordination of therapeutic service providers ensures efficient mobility of people with their necessary health and everyday activities. - Therapeutic mobilities come to the aid of bodies that need diagnostics, therapies or palliative care, thus combining movement and stasis to unfold therapeutic capacities. - The transnational trade in traditional medicines exerts therapeutic effects on the communities engaged in these commercial exchanges, not just those using the medicines. - Nurses provide therapy, yet in exercising mobility the profession of nursing is itself enhanced. |

80

| Title: Blue space geographies: Enabling health in place |
| --- |
| Year of publication: 2015 |
| Type of literature: Journal article |
| Journal name: Health & Place |
| Name of authors: Ronan Foley, Thomas Kistemann |
| Peer-reviewed: Yes |
| Extracted findings:   - The confluence of physical and social conditions occurs not only in ‘green’ or ‘blue’ spaces but also in commercial settings such as a grocery store, thus suggesting that blue spaces can have the same restoration potential as a part of the natural settings. - The role of blue space is evident in improving people`s health and well-being. - Blue space attractions, such as water flow, are associated with higher preferences and more positive subjective reactions of visitors of landscapes. |

81

| Title: Baltic Health Tourism: Uniqueness and Commonalities |
| --- |
| Year of publication: 2015 |
| Type of literature: Journal article |
| Journal name: Scandinavian Journal of Hospitality and Tourism |
| Name of authors: Melanie Smith |
| Peer-reviewed: Yes |
| Extracted findings:   - Regional and national collaboration is a crucial prerequisite for health tourism destination development offering health and wellness treatments. - Health tourism is highly fragmented and diverse and it encompasses different meanings of health treatments, tourism, supporting service industries, and the quality of natural and built environment to attract tourists. |

82

| Title: Health resorts and multi-textured perceptions of international health tourists |
| --- |
| Year of publication: 2018 |
| Type of literature: Journal article |
| Journal name: Sustainability |
| Name of authors: Majeed, S., Lu, C., Majeed, M., and Shahid, M. N. |
| Peer-reviewed: Yes |
| Extracted findings:   - It is a challenging task to read and shape the positive behavioral intentions of health tourists, and attract them towards the health resorts having different combinations of natural and built attractions. - Complex relationships between the perceived attractions of a health resort, health tourists’ expectations, and their behavioral intentions demand in-depth analysis from the stakeholders of health resorts. - Deeper and broader associations among the stakeholders of health, medical, wellness, and sustainable tourism industries are needed which merge in health resorts in order to deliver a satisfied health care experience. |

83

| Title: The journey from an allopathic to natural treatment approach: A scoping review of medical tourism and health systems |
| --- |
| Year of publication: 2017 |
| Type of literature: Journal article |
| Journal name: European Journal of Integrative Medicine |
| Name of authors: Majeed, S., Lu, C., and Javed, T. |
| Peer-reviewed: Yes |
| Extracted findings:   - Modern concepts of medicine, health, and tourism have gone beyond ordinary treatment concepts due to changing preferences of medical tourists. - The health industry is experiencing soft medical tourism demands from health and well-being seeking people which are grounded in natural treatments in natural and built environments alongside the attractions of alternative health treatments, such as T & CM. - Appropriate connections between allopathic and natural treatment providers may ensure tourists’ health and well-being at the potential healing centers located at tourist destinations. |

84

| Title: The impact of blue space on human health and well-being-Salutogenetic health effects of inland surface waters: A review |
| --- |
| Year of publication: 2011 |
| Type of literature: Journal article |
| Journal name: International Journal of Hygiene and Environmental Health |
| Name of authors: Völker, S., and Kistemann, T. |
| Peer-reviewed: Yes |
| Extracted findings:   - Blue space has manifold influences on human health and wellbeing. - Emotional and experiential responses to blue space need to be recognized well. - It is suggested that introducing ‘blue’ as a new colour (both literally and metaphorically) into debates on environmental health and therapeutic landscapes will ensure people’s desire for optimal health and well-being. |

85

| Title: Tourists and severe weather-An exploration of the role of ‘locus of responsibility’ in protective behaviour decisions |
| --- |
| Year of publication: 2013 |
| Type of literature: Journal article |
| Journal name: Tourism Management |
| Name of authors: Jeuring, J., and Becken, S. |
| Peer-reviewed: Yes |
| Extracted findings:   - Tourist safety is critical for destinations to achieve high levels of satisfaction, avoid accidents and minimise negative publicity. - Collaborations between scientists and the industry professionals may help to promote the positive image of destinations. - Tourism-specific campaigns should focus on providing hazard specific knowledge to increase protection efficacy amongst tourists, but they should also enhance tourists’ perceptions of responsibility for self-protection, including information on which organisations are responsible for particular aspects of safety. |

86

| Title: Desert as therapeutic space: Cultural interpretation of embodied experience in sand therapy in Xinjiang, China |
| --- |
| Year of publication: 2018 |
| Type of literature: Journal article |
| Journal name: Health & Place |
| Name of authors: Wang, K., Cui, Q., and Xu, H. |
| Peer-reviewed: Yes |
| Extracted findings:   - Deserts act as a therapeutic landscape. - Hot sand and the natural desert environment offer tactile stimulation, causing people have the sensory experiences of burning, heat and sweating. - Influenced by cultural beliefs, the tactile sensations in deserts were constructed as both means to obtain and symptom of therapeutic effects, which fulfilled people’s imaginations and expectations of a therapeutic space. |

87

| Title: Changing preferences, moving places and third party administrators: A scoping review of medical tourism trends (1990-2016) |
| --- |
| Year of publication: 2017 |
| Type of literature: Journal article |
| Journal name: Almatourism-Journal of Tourism, Culture and Territorial Development |
| Name of authors: Majeed, S., Lu, C. |
| Peer-reviewed: Yes |
| Extracted findings:   - Tourists’ prefer to avail a variety of health treatments at tourism places covered with natural and built environments. - Tourists’ health and well-being is based on satisfaction of finding desired health treatments. - Third party administrators and other service providers may ensure the provision of desired health treatments, such as conventional medical treatments and T&CM, to health-seeking tourists. |

88

| Title: Disaster tourism and disaster landscape attractions after Hurricane Katrina: An auto-ethnographic journey |
| --- |
| Year of publication: 2008 |
| Type of literature: Journal article |
| Journal name: International Journal of Culture, Tourism and Hospitality Research |
| Name of authors: Miller, D. S. |
| Peer-reviewed: Yes |
| Extracted findings:   - The tour, marked with an over arching sense of loss, shock, and dread, negatively influence visitors’ perceptions and behaviors. - Disaster landscapes reflect the bad image of destination. - After environmental changes at a destination after crises, the negative images of the devastation can be improved with advertisement to positively influence the decision making of tourists. |

89

| Title: Environment as a key factor of health and well-being tourism destinations in five European countries |
| --- |
| Year of publication: 2019 |
| Type of literature: Journal article |
| Journal name: IBIMA Business Review |
| Name of authors: Plzáková, L., and Stupková, C. |
| Peer-reviewed: Yes |
| Extracted findings:   - Motivations facilitate the formation of attachment to a large natural environment. - Place-based” motivations are the perceived benefits that emerge from opportunities afforded by an environment. - Environmental worldviews underpin and help to explain the linkage between motivation and place attachment. - Everyday multisensory environments impact feelings of safety and happiness of people |

90

| Title: Small health pilgrimages: Place and practice at the holy well |
| --- |
| Year of publication: 2013 |
| Type of literature: Journal article |
| Journal name: Culture and Religion |
| Name of authors: Foley, R. |
| Peer-reviewed: Yes |
| Extracted findings:   - Places, holy wells, improve people`s health and well-being. - Holy wells reflect healing landscapes that show how dynamic material, social, cultural, and personal assemblages entwine to shape a sense of wellbeing. - Holy well offers spiritual treatments with social interaction with other people that ensures visitors’ health and well-being. |

91

| Title: Preparation for crisis management: A proposed model and empirical evidence |
| --- |
| Year of publication: 2004 |
| Type of literature: Journal article |
| Journal name: Journal of Contingencies and Crisis Management |
| Name of authors: Elsubbaugh, S., Fildsen, R., and Rosenn, M. B. |
| Peer-reviewed: Yes |
| Extracted findings:   - The effective management of crises improves destination image. - Crisis management helps to minimize and manage the impacts of crisis on visitors’ physical and mental health. - Crisis management strategies may keep existing visitors alongside attracting new visitors. |

92

| Title: Segmentation by push motives in health tourism destinations: A case study of polish spa resort |
| --- |
| Year of publication: 2018 |
| Type of literature: Journal article |
| Journal name: Journal of Destination Marketing & Management |
| Name of authors: Dryglas, D., and Salamaga, M. |
| Peer-reviewed: Yes |
| Extracted findings:   - Alternative treatments, such as spa, may improve people’s health and well-being. - Staying at tourism destination for healing and recuperation or for alternative health treatments such as massage and spa, may hold tourists’ interests with satisfaction. - Spa makes tourists feel relaxed, improve mind, body, and soul, and perform better in daily activities. - Healing centers with alternative therapies, e.g., a spa resort, have healing impacts on the body, mind, and spirit of visitors. - Spa treatments reflect people’s lifestyle and preferences for a better quality of life. |

93

| Title: Dry cupping therapy and the wellness management of health travelers |
| --- |
| Year of publication: 2019 |
| Type of literature: Journal article |
| Journal name: Traditional Medicine Research |
| Name of authors: Majeed, S., Majeed, M., and Ajike, M.A. |
| Peer-reviewed: Yes |
| Extracted findings:   - Health perceptions with the sub-constructs of physical feelings and psychological feelings are significantly related to the health status of individuals - Alternative therapies at built environment help to improve tourists’ health and well-being. - Positive improvements are observed for general physical and mental health, emotions and expressions, relaxation, quality of sleep, inner peace, and satisfaction after the intervention of alternative health treatments at therapy centers built for people’s health and well-being. |

94

| Title: Beauty and elegance: Value co-creation in cosmetic surgery tourism |
| --- |
| Year of publication: 2020 |
| Type of literature: Journal article |
| Journal name: SAGE Open |
| Name of authors: Salman Majeed, Zhimin Zhou, Haywantee Ramkissoon |
| Peer-reviewed: Yes |
| Extracted findings:   - The notions of medical tourism, health, and well-being are interlinked. - Conventional and alternative health treatments help to meet tourists’ expectations of optimal health and well-being. - Tourist satisfaction about the available wellness treatments in natural and built environments ensures their repeat visits. |

95

| Title: Crisis management: A suggested typology |
| --- |
| Year of publication: 2020 |
| Type of literature: Journal article |
| Journal name: Journal of Travel and Tourism Marketing |
| Name of authors: Laws, E., and Prideaux, B. |
| Peer-reviewed: Yes |
| Extracted findings:   - An event, in whatever form it occurs, that creates a shock to the tourism industry resulting in the sudden emergence of an adverse situation, is usually referred to as a crisis. - A crisis is usually characterized by falling visitor numbers followed by a fall in employment, decline in private sector profits and eventually a reduction in government revenue, and possibly cessation of further investment. |

96

| Title: The perceived impact of risks on travel decisions |
| --- |
| Year of publication: 2006 |
| Type of literature: Journal article |
| Journal name: International Journal of Tourism Research |
| Name of authors: Law, R. |
| Peer-reviewed: Yes |
| Extracted findings:   - Reduced perceived risk of destination crisis attracts tourists to destinations. - People feel dissatisfaction due to perceived risk. - Tourists’ decisions to travel are susceptible to the level of perceived risk at host destination. |

97

| Title: Crises” that scare tourists: Investigating tourists’ travel-related concerns |
| --- |
| Year of publication: 2007 |
| Type of literature: Book chapter |
| Book title: Crisis Management in Tourism |
| Name of authors: Dolnicar S. |
| Peer-reviewed: Yes |
| Extracted findings:   - Destination crises fuel perceived risk and discourage tourist’ from crisis-hit destinations. - The effective management of crises is a strategic necessity to reduce perceived risk of any tourism destination. - Perceived risk of destination crises exert negative influence on tourists’ physical and mental health. - Reduced perceived risk of destination crisis attracts tourists to destinations. |

98

| Title: What tourists worry about-construction of a scale measuring tourist worries |
| --- |
| Year of publication: 2009 |
| Type of literature: Journal article |
| Journal name: Tourism Management |
| Name of authors: Larsen, S., Brun, W., and Øgaard, T. |
| Peer-reviewed: Yes |
| Extracted findings:   - Tourists’ prefer to travel to destinations which are less risky. - Tourists’ decisions to travel to tourism destinations depend on different types of risks, such as financial and health risk. - Risk management strategies and communication with potential tourists’ significantly influence perceived image of destinations. |

99

| Title: The relationships among adult sustainability attitudes, psychological well-being, nature relatedness, and interest in scientific issues |
| --- |
| Year of publication: 2020 |
| Type of literature: Journal article |
| Journal name: Current Psychology |
| Name of authors: Wang, H.-H., Hong, Z.-R., Lin, H.-S., and Tsai, C.-Y. |
| Peer-reviewed: Yes |
| Extracted findings:   - Good natural environment people’s health and well-being. - The quality of environment helps to develop sustainable attitudes of the residents and the visitors of places. - Good natural environment helps to develop mental and physical well-being of people alongside positive sustainable attitudes. |

100

| Title: Restoring tourism destination in Crisis: A strategic management approach |
| --- |
| Year of publication: 2003 |
| Type of literature: Book |
| Publisher: Allen & Urwin, Australia |
| Name of authors: Beirman |
| Reviewed by Publisher: Yes |
| Extracted findings:   - Tourism destination crises may involve financial, health, and psychological risk. - Destination crises negatively impact tourists’ arrival. - Tourists’ psychological health is largely determined by the perceived risk of destination crises. - Effective crisis management strategies may help to improve the destination during and after crises. |

101

| Title: Middle East respiratory syndrome coronavirus (MERs-Cov): Prevention in travelers |
| --- |
| Year of publication: 2014 |
| Type of literature: Journal article |
| Journal name: Travel Medicine and Infectious Disease |
| Name of authors: Pavli, A., Tsiodras, S., and Maltezou, H. C. |
| Peer-reviewed: Yes |
| Extracted findings:   - Disease outbreak discourages travelers from visiting the destinations. - Travelers’ are highly susceptible to catching infectious diseases. - Destinations with epidemics, such as MERs-Cov, bear long lasting consequences in terms of tourist arrivals. - Effective pandemic management may reduce the risk of catching infectious disease at early stage. |

102

| Title: The impacts of SARS on Hong Kong’s tourism industry |
| --- |
| Year of publication: 2004 |
| Type of literature: Journal article |
| Journal name: International Journal of Contemporary Hospitality Management |
| Name of authors: Pine, R., and McKercher, B. |
| Peer-reviewed: Yes |
| Extracted findings:   - The tourism industry is the most volatile industry among all other service industries. - SARS pandemic impacted global tourism and endangered public health and well-being. - SARS reduced tourists’ demands due to perceived risk of catching infectious disease. - Epidemics negatively influence visitors’ and residents’ health and well-being. |

103

| Title: An earthquake disaster management mechanism based on risk assessment information for the tourism industry-a case study from island of Taiwan |
| --- |
| Year of publication: 2010 |
| Type of literature: Journal article |
| Journal name: Tourism Management |
| Name of authors: Tsi, C.-H., and Chen, C.-W. |
| Peer-reviewed: Yes |
| Extracted findings:   - Effective risk management strategies may help tourists’ safety. - Sharing relevant and timely information with tourists may help to promote the positive image of the destination during and after crises. - Information sharing is an important tool to positively influence tourists’ perceptions of the destination. |

104

| Title: Access to the outdoors: using photographic comparison to assess preferences of assisted living residents |
| --- |
| Year of publication: 2005 |
| Type of literature: Journal article |
| Journal name: Landscape and Urban Planning |
| Name of authors: Rodiek, S. D., and Fried, J. T. |
| Peer-reviewed: Yes |
| Extracted findings:   - Features depiction is an important tool in designing for outdoor usage at long-term care facilities. - Healing places may ensure mental and physical health of people with “more places to walk”, “more trees”, and “benches to rest”. |

105

| Title: Wellness tourism management: Well-being as a sustainability concern for wellness tourism management |
| --- |
| Year of publication: 2020 |
| Type of literature: Book chapter |
| Book title: Industrial and Managerial Solutions for Tourism Enterprises |
| Name of authors: Sonuç, N. |
| Peer-reviewed: Yes |
| Extracted findings:   - Wellness tourists prefer to avail a variety of wellness treatments and tourism to find optimal health and well-being. - Wellness activities, such as yoga, in natural environment offer optimal health and well-being to tourists. - Sustainable tourism attractions are helpful to improve tourists’ health and well-being. |

106

| Title: Effective crisis management: Worldwide principles and practice |
| --- |
| Year of publication: 2000 |
| Type of literature: Book |
| Publisher: Casell, London |
| Name of authors: Seymour, M., and Moore, S. |
| Reviewed by Publisher: Yes |
| Extracted findings:   - Destination crises fuel people’s risk perceptions. - Crises discourage people from visiting places. - Crisis-hit destinations may face fall in their economic share. - Crises exert long-lasting impacts on the reputation of destinations. - Effective crises management strategies may promote positive image of the destination alongside attracting visitors. |

107

| Title: Therapeutic landscapes, restorative environments, place attachment, and well-being |
| --- |
| Year of publication: 2018 |
| Type of literature: Book chapter |
| Book title: Oxford Textbook of Nature and Public Health: The role of Nature in Improving the Health of a population |
| Name of authors: Townsend, M., Henderson-Wilson, C., Ramkissoon, H., and Werasuriya, R. |
| Peer-reviewed: Yes |
| Extracted findings:   - Therapeutic landscapes provide restorative environments to the visitors. - Natural environment of therapeutic landscapes offers health and well-being to visitors. - The restorative properties of therapeutic landscapes may keep visitors’ loyalty, such as repeat visits. - Visitors’ positive perceptions about the restorative properties of therapeutic landscapes impact their mental and physical health for optimal quality of life. |

108

| Title: Social involvement and park citizenship as moderators for quality-of-life in a national park |
| --- |
| Year of publication: 2018 |
| Type of literature: Journal article |
| Journal name: Journal of Sustainable Tourism |
| Name of authors: Ramkissoon, H., Mavondo, F., and Uysal, M. |
| Peer-reviewed: Yes |
| Extracted findings:   - Parks offer opportunities of social interaction with other people. - Social interaction impact positively on people’s health and well-being. - Parks with built settings improve quality of life of people. - Well-being and enhanced quality-of-life induced by visiting natural settings is an important outcome for the individual and for the society. - Visitors’ place satisfaction is a critical antecedent to enhancing one’s quality of experience, thus its contribution to quality-of-life. |

109

| Title: Relationships between place attachment, place satisfaction and pro-environmental behaviour in an Australian national park |
| --- |
| Year of publication: 2013 |
| Type of literature: Journal article |
| Journal name: Journal of Sustainable Tourism |
| Name of authors: Ramkissoon, H., Smith, L. D. G., and Weiler, B. |
| Peer-reviewed: Yes |
| Extracted findings:   - There is a positive relationship with place satisfaction and pro-environmental behavioural intentions. - Place dependence, place identity, place social bonding and place affect have different psychological properties and their effects on place satisfaction and proenvironmental behaviours are important to explore for visitors’ health and well-being. - Visitors’ place attachment shows multi-faceted dimensions such as cognitions, identity, emotions, and affect. - place dependence, place identity, place social bonding and place affect are significant determinants of visitor satisfaction with the park and that some were significantly related to both low and high effort pro-environmental behavioural intentions. |

110

| Title: Testing the dimensionality of place attachment and its relationship with place satisfaction and pro-environmental behaviours: A structural equation modeling approach |
| --- |
| Year of publication: 2013 |
| Type of literature: Journal article |
| Journal name: Tourism Management |
| Name of authors: Ramkissoon, H., Smith, L. D. G., and Weiler, B. |
| Peer-reviewed: Yes |
| Extracted findings:   - Visitors who are generally satisfied with their decision to visit the park may not see the need to improve the park’s environment, perhaps because the park and its resources are already meeting their visitation goals and are perceived to be in an optimal condition by such visitors. - Visitors may not find any need to enhance the environment by engaging in environmental behaviours that involve high effort, but are willing to engage in those environmental behaviours that involve low efforts to maintain and protect the park’s environment. - Visitors’ attachment to the park may be improved with distinctive attributes, such as infrastructure, affective components, and activities. |

111

| Title: Place attachment and pro-environmental behaviour in national parks: the development of a conceptual framework |
| --- |
| Year of publication: 2012 |
| Type of literature: Journal article |
| Journal name: Journal of Sustainable Tourism |
| Name of authors: Ramkissoon, H., Weiler, B., and Smith, L. D. |
| Peer-reviewed: Yes |
| Extracted findings:   - Visitors are satisfied with their decision to visit the park due to the restorative combinations in natural and built setting. - Visitors’ place attachment with natural and built settings, such as parks and community gardens, reflects their satisfaction. - Visitors’ health and well-being is determined by the restorative properties of parks which lead to place attachment. - Visitors’ positive emotions and feelings of improvement in the health lead to their place attachment. |

112

| Title: Tourism growth and regional resilience: The ‘beach disease’ and the consequences of the global crisis of 2007 |
| --- |
| Year of publication: 2016 |
| Type of literature: Journal article |
| Journal name: Tourism Economics |
| Name of authors: Romao, J., Guerreiro, J., and Rodrigues, P. |
| Peer-reviewed: Yes |
| Extracted findings:   - Disease outbreaks, such as beach disease, impact the national and regional economy, leading to a significant decrease of tourism in the region. - Tourists’ health and well-being concerns become more important in the event of destination crises, such as disease outbreaks. - Slump in tourism demand is observed by the destinations exposed to disease outbreaks. |

113

| Title: The severe acute respiratory syndrome: Impact on travel and tourism |
| --- |
| Year of publication: 2006 |
| Type of literature: Journal article |
| Journal name: Travel Medicine and Infectious Disease |
| Name of authors: Wilder-Smith, A. |
| Peer-reviewed: Yes |
| Extracted findings:   - The international spread of disease underscores the need for strong global public health systems, excellent international reporting mechanisms, robust health service infrastructures, and expertise that can be mobilized quickly across national boundaries to mirror disease movements. - The psychological impacts of SARS, coupled with travel restrictions imposed by various national and international authorities, have diminished international travel. - Governments and press, especially in non SARS affected areas, have been slow to strike the right balance between timely and frequent risk communication and placing risk in the proper context - Alerts or bulletins to provide accurate information about the status of SARS at a destination with travel advisories against nonessential travel to the area help to boost destination image during and after the pandemic. |

114

| Title: Tourism and the health effects of infectious diseases: Are there potential risks for tourists? |
| --- |
| Year of publication: 2015 |
| Type of literature: Journal article |
| Journal name: International Journal of Safety and Security |
| Name of authors: Baker, D.M.A |
| Peer-reviewed: Yes |
| Extracted findings:   - The risk of catching infectious diseases negatively impact tourists’ behaviors, such as satisfaction, intentions to visit. - The health and well-being notions of potential tourist destinations are considered in the context of tourists’ safety from financial, physical, and health threats. - The potential threats of infectious disease discourage tourists’ from visiting tourist destinations. |

115

| Title: Potentials of tourism products and services in Bangladesh |
| --- |
| Year of publication: 2020 |
| Type of literature: Book chapter |
| Book title: Tourism Marketing in Bangladesh |
| Name of authors: Hassan, A., and Ramkissoon, H. |
| Peer-reviewed: Yes |
| Extracted findings:   - Staying at leisure settings with exposure to sun and fun activities, sauna, massage, spa, and other health activities promote visitors’ health and well-being. - Sustainable places with natural and built environments positively influence tourists` psychological filters. - Tourists` well-being are susceptible to the quality of natural environment. - Recreational facilities with social gathering exert positive impacts on tourists` everyday behaviors. |

116

| Title: Shinrin-Yoku (Forest Bathing) and Nature Therapy: A state-of-the-Art Review |
| --- |
| Year of publication: 2017 |
| Type of literature: Journal article |
| Journal name: International Journal of Environmental Research and Public Health |
| Name of authors: Hansen, M. M., Jones, R., and Tocchini, K. |
| Peer-reviewed: Yes |
| Extracted findings:   - Human physiological/psychological functions are deeply-rooted in the nature. - Advancements in alternative medical treatments have promoted people’s health and well-being without potential side effects of treatments and recuperation in the natural environment. - Psychologically and spiritually speaking, humans intuitively know the relaxing, soothing and “awe” effects of being in or viewing forests, plants, flowers, urban green spaces, parks and natural wooden material. |

117

| Title: The relationship between place attachment and landscape values: Toward mapping place attachment |
| --- |
| Year of publication: 2007 |
| Type of literature: Journal article |
| Journal name: Applied Geography |
| Name of authors: Brown, G., and Raymond, C. |
| Peer-reviewed: Yes |
| Extracted findings:   - An abundance of aesthetic and wilderness/natural landscape features, along with recreation and therapeutic experiences in those landscapes, help create the conditions that lead to place attachment - It is the individual’s willingness to associate spiritual value with a landscape that best predicts the psychological state of place attachment. - The spatial analysis of landscape value densities with the map-based measure of place attachment (special places) confirms the importance of human engagement with a landscape in developing some level of place attachment. - The significant spatial co-location of recreation and aesthetic values with special place locations, and to a lesser extent, economic and therapeutic values, reflect the transactive nature of human–landscape interactions. |

118

| Title: The satisfaction-place attachment relationship: Potential mediators and moderators |
| --- |
| Year of publication: 2015 |
| Type of literature: Journal article |
| Journal name: Journal of Business Research |
| Name of authors: Ramkissoon, H., and Mavondo, F.T. |
| Peer-reviewed: Yes |
| Extracted findings:   - Person-place bonding affects preferences and the perception of place restorative properties - Place attachment consistently and positively predicted the assessment of the restorative properties of healthy places. - Place attachment is an emotional bond that people establish with significant places; that is, places they visit or use regularly and that make them feel at ease and satisfied. |

119

| Title: Post-SARS tourist arrival recovery patterns: an analysis based on a catastrophe theory |
| --- |
| Year of publication: 2010 |
| Type of literature: Journal article |
| Journal name: Tourism Management |
| Name of authors: Mao, C.-K., Ding, C. G., and Lee, H.-Y. |
| Peer-reviewed: Yes |
| Extracted findings:   - Macro strategy targeted to the general public: to enhance the travelers’ confidence regarding the safety of the destination country through the mass media campaign to improve the public perception in the origin country - The promotion campaigns conducted in different origin countries had affected their public and individual perceptions that ultimately impact their mental health. - Tourism demand is particularly sensitive to security and health concerns, and the industry is highly susceptible to changes in the international political situation, natural disasters, and epidemics. |

120

| Title: Post-SARS tourist arrival recovery patterns: an analysis based on a catastrophe theory |
| --- |
| Year of publication: 2010 |
| Type of literature: Journal article |
| Journal name: Tourism Management |
| Name of authors: Mao, C.-K., Ding, C. G., and Lee, H.-Y. |
| Peer-reviewed: Yes |
| Extracted findings:   - Macro strategy targeted to the general public: to enhance the travelers’ confidence regarding the safety of the destination country through the mass media campaign to improve the public perception in the origin country - The promotion campaigns conducted in different origin countries had affected their public and individual perceptions that ultimately impact their mental health. - Tourism demand is particularly sensitive to security and health concerns, and the industry is highly susceptible to changes in the international political situation, natural disasters, and epidemics. |

121

| Title: Perceptions of importance and what safety is enough |
| --- |
| Year of publication: 2012 |
| Type of literature: Journal article |
| Journal name: Journal of Business Research |
| Name of authors: |
| Peer-reviewed: Yes |
| Extracted findings:   - Due to the increased threats of disease and natural disasters, tourism service providers are taking safety and security actions to protect the health and well-being of tourists. - In pandemics, such as Severe Acute Respiratory Syndrome (SARS) outbreak, a destination’s effective management to control pandemic spread, e.g., to issue face masks at airports, tourists go through thermal detectors, may help to boost tourists’ confidence on the destination. - Risk taking is different from recognizing risk in making decisions. - Tourists pay attention to security when traveling and choose travel destinations not only on the basis of price. |

122

| Title: Willingness to take travel-related health risks-A study among Finnish tourists in Asia during the Avian Influenza Outbreak |
| --- |
| Year of publication: 2009 |
| Type of literature: Journal article |
| Journal name: International Journal of Behavioral Medicine |
| Name of authors: Aro, A. R., Vartti, A.-M., Schreck, M., Turtiainen, P., and Uutela, A. |
| Peer-reviewed: Yes |
| Extracted findings:   - Different travelers take travel-related health risks differently. - Different kinds of travelers are exposed to different kinds of risk which further impact their mental and physical health differently. - Holidays more often pose risks related to “letting it go,” hedonistic life style, and looking for experiences, which increase the risk of infectious diseases such as HIV, food-related infections, and accidents |

123

| Title: Place satisfaction, place attachment and quality of life: development of a conceptual framework for island destination |
| --- |
| Year of publication: 2016 |
| Type of literature: Book chapter |
| Book title: *Sustainable Island Tourism: Competitiveness and quality of life* |
| Name of authors: Ramkisson, H. |
| Peer-reviewed: Yes |
| Extracted findings:   - Island tourism offers the opportunities of health and well-being to tourists. - Tourists’ quality of life improves after visiting islands with abundant natural and built resources. - Tourist revisits to destinations are based on their feeling of improvement in health and well-being. |

124

| Title: Proenvironmental behavior: Critical link between satisfaction and place attachment in Australia and Canada |
| --- |
| Year of publication: 2017 |
| Type of literature: Journal article |
| Journal name: Tourism Analysis |
| Name of authors: Ramkissoon, H., & Mavondo, F. |
| Peer-reviewed: Yes |
| Extracted findings:   - Proenvironmental behavior as a tool for enhancing sustainability of tourist attractions. - For visitors in search of distinctive natural scenery and cultural assets, parks having abundant natural settings often hold special meanings and, as such, facilitate social and psychological interactions between people and settings for overall health and well-being. - These interactions often result in high levels of place satisfaction and become visitors’ favorite places by positively influencing their perceptions. |
