# Supplementary material for: Health, Wellness, and Place Attachment During and Post Health Pandemics
Source: Front Psychol. 2020 Nov 26;11:573220. doi: 10.3389/fpsyg.2020.573220 (PMC7726477; doi:10.3389/fpsyg.2020.573220)
Supplement: Supplementary file 2 [file Table_2.docx]

**Appendix 2**

**Table 2** Literature review summary

| **Author (s)** | **Year** | **Main notes** |  | | **Extracted themes** | | | | | | | | |
| --- | --- | --- | --- | --- | --- | --- | --- | --- | --- | --- | --- | --- | --- |
|  |  |  | PGTL | NL | | BL | LD | HM | TN | VDTL | HWB | PA | DC |
| Ramkissoon | 2014 | Spa; wellness retreat; therapeutic places | X | X | | X | X |  | X | X | X | X |  |
| van Riper et aL. | 2019 | Nature; place attachment | X | X | |  | X |  |  | X | X | X |  |
| Colley and Craig | 2019 | Natural places; individuals’ perceptions; place attachment | X | X | |  | X |  |  | X | X | X |  |
| Menatti et al. | 2019 | Place attachment; landscape restorativeness | X | X | | X | X |  |  | X | X | X |  |
| Gössling et al. | 2020 | Pandemic; tourism; COVID-19 | X |  | |  | X | X | X | X | X | X | X |
| Kale | 2019 | Place attachment; Well-being | X | X | |  |  |  |  | X | X | X |  |
| Twedt et al. | 2019 | Nature and perceived restorativeness; visual appeal | X | X | |  | X |  |  | X | X | X |  |
| Rosenbaum et al. | 2020 | Therapeutic landscapes | X | X | |  | X | X |  | X | X | X |  |
| Martin et al. | 2020 | Nature, health, and well-being; pro-environmental behavior | X | X | | X | X | X |  | X | X | X |  |
| Bugden and Stedman | 2019 | Place and behavior | X |  | |  |  |  |  | X | X | X |  |
| Park and Mattson | 2009 | Indoor plants and hospital’s internal environment; patients’ health and well-being | X |  | | X |  | X |  | X | X | X |  |
| MacKenzie and Brymer | 2020 | Nature sport; nature and health | X | X | |  | X |  |  | X | X |  |  |
| Yeo et al. | 2020 | Internal environmental settings for health and well-being | X |  | | X |  | X |  | X | X |  |  |
| Coburn et al. | 2019 | Psychological responses and behaviors, natural environment | X | X | | X | X |  |  |  | X | X | X |
| Jiang et al. | 2016 | Destination image; place attachment | X | X | | X | X |  |  | X | X | X |  |
| Bolten et al. | 2020 | Built environment; physical and psychological health and well-being | X | X | | X | X | X |  | X | X | X |  |
| Tse | 2006 | Tourism; crisis; destination and landscape | X |  | |  | X |  |  |  | X |  | X |
| Jiang et al. | 2018 | Natural soundscapes; nature and tourism | X | X | | X | X | X | X | X | X | X |  |
| Harvey et al. | 2020 | Biodiversity; psychological benefits | X | X | | X |  | X |  | X | X |  |  |
| Plane and Klodawsky | 2013 | Health and well-being; place and health; park | X |  | | X | X |  |  | X | X |  |  |
| Andrews et al. | 2005 | Nursing home geographies | X |  | | X |  |  | X | X | X |  |  |
| Andrews | 2004 | Health care and place; therapeutic geographies | X | X | | X | X |  | X | X | X |  |  |
| Smith and Puczkó | 2014 | Wellness treatment; medical travel; health and well-being | X |  | | X | X | X |  | X | X |  |  |
| Choe et al. | 2020 | Natural environment; health and well-being | X | X | | X |  | X |  | X | X |  |  |
| Smith and Puczko | 2009 | Health and wellness tourism; place and health | X | X | | X |  |  |  | X | X |  |  |
| Karmanov and Hamel | 2008 | Therapeutic landscape; urban environment | X | X | |  | X |  |  | X | X |  |  |
| Oster et al. | 2011 | Therapeutic landscapes: health and well-being | X | X | | X | X |  |  | X | X |  |  |
| Wang et al. | 2020 | Psychology; behaviors; disease outbreak; COVID-19 | X |  | |  |  | X |  | X | X | X | X |
| Yamashita | 2002 | Perceptions of landscapes; blue space; natural environment | X | X | |  | X | X |  | X | X |  |  |
| Wilton and DeVerteuil | 2006 | Health and place; therapeutic landscape | X |  | | X |  |  |  | X | X |  |  |
| Milligan et al. | 2004 | Therapeutic landscapes; aging population | X | X | |  | X |  |  | X | X |  |  |
| Maxfield and Wiltshier | 2019 | Water festivals; visitors’ motivations | X | X | |  | X |  |  | X | X | X |  |
| Hall and Page | 2002 | Geography; environment; health and place | X | X | | X | X | X |  | X | X |  |  |
| White et al. | 2010 | Natural and built scenes; blue space | X | X | | X | X |  | X | X | X |  |  |
| Asakawa et al. | 2004 | Green places; urban planning | X | X | | X | X |  |  | X | X |  |  |
| Pitt | 2014 | Therapeutic landscape; community gardens | X | X | | X | X |  |  | X | X |  |  |
| Steinwender et al. | 2008 | Water; blue space; land and water | X | X | |  | X |  |  | X | X |  |  |
| Gesler | 2003 | Healing and recovery; place, health, and well-being | X | X | | X | X | X | X | X | X |  |  |
| Britton et al. | 2020 | Blue space; health and well-being | X | X | |  | X | X |  |  | X | X |  |
| Stigsdotter et al. | 2010 | Nature-based therapeutic interventions; health and place | X | X | | X | X | X |  | X | X |  |  |
| Dustin et al. | 2010 | People-nature interaction; place and health; health promotion | X | X | | X | X |  |  | X | X |  |  |
| Hoffman | 2017 | Pharmaceuticals; tourist spaces; therapeutic landscapes; health and place | X | X | | X | X |  |  | X | X |  |  |
| Sampson and Gifford | 2010 | Place-making; well-being; therapeutic landscapes; role of place for healing and recovery | X | X | |  |  |  |  | X | X |  |  |
| Nunkoo and Ramkissoon | 2013 | Enclave tourism; tourism stakeholders | X | X | |  | X | X | X | X | X | X |  |
| Rose | 2012 | Therapeutic landscapes; psychoanalytic analysis; space between mind and place | X |  | |  |  |  |  | X | X |  |  |
| Kennedy et al. | 2004 | Therapeutic places; health and well-being |  | X | | X | X |  |  | X | X |  |  |
| Agyekum and Newbold | 2016 | Spirituality; therapeutic landscape; health and place | X | X | | X | X |  |  | X | X |  |  |
| Bone | 2013 | Spiritual retreat tourism; wellness tourism; therapeutic destination | X | X | | X | X |  |  | X | X |  |  |
| Williams | 2010 | Spiritual therapeutic landscapes; place and healing | X | X | | X | X |  |  | X | X |  |  |
| Williams | 2013 | Therapeutic landscapes; cyberpilgrimage | X |  | | X | X |  |  | X | X |  |  |
| Evans et al. | 2009 | Therapeutic landscapes; therapeutic aesthetics; medical spaces | X | X | | X | X |  |  | X | X |  |  |
| Buzinde and Yarnal | 2012 | Therapeutic landscapes; medical tourism | X | X | | X |  |  | X | X | X |  |  |
| Bell et al. | 2018 | Therapeutic landscape: healthy places | X | X | | X | X |  |  | X | X |  |  |
| Dunkley | 2009 | Therapeutic landscape; wilderness therapy; health and place | X | X | | X | X |  |  | X | X |  |  |
| Huang and Xu | 2018 | Therapeutic landscape; health and place | X | X | | X | X |  |  | X | X |  |  |
| Smyth | 2005 | Therapeutic landscapes; health and well-being | X |  | |  | X |  | X | X | X |  |  |
| Finlay et al. | 2015 | Therapeutic landscapes; well-being; aging population; blue and green places | X | X | | X | X | X |  | X | X |  |  |
| Moon et al. | 2006 | Therapeutic landscapes | X | X | | X | X |  |  | X | X |  |  |
| Gatrell | 2013 | Therapeutic landscape; health and well-being | X | X | |  | X | X |  | X | X |  |  |
| Novelli et al. | 2018 | Disease outbreak; tourism crisis; Health and well-being | X |  | |  |  | X |  | X | X |  | X |
| Smith and Diekmann | 2017 | Tourism; health and well-being; therapeutic landscapes | X | X | | X | X |  |  | X | X |  |  |
| Bell et al. | 2015 | Health and well-being; water, land, blue space; therapeutic landscape | X | X | |  |  |  |  | X | X |  |  |
| Bell et al. | 2017 | Therapeutic landscapes; health and place; green space; blue space | X | X | | X | X | X | X | X | X |  |  |
| Little | 2013 | Therapeutic landscapes; well-being; women health | X | X | |  |  | X |  | X | X |  |  |
| Love et al. | 2012 | Therapeutic landscape; drug treatment; place and health | X |  | | X |  |  | X |  | X |  |  |
| Hoyez | 2007 | Yoga; therapeutic landscape; health and place | X | X | | X |  | X |  | X | X |  |  |
| Williams | 2014 | Health and place; place and healing | X | X | | X | X | X |  | X | X |  |  |
| Völker and Kistemann | 2013 | Water, land, health, and well-being; therapeutic landscape | X | X | |  | X |  |  | X | X |  |  |
| Coghlan | 2015 | Tourism and health; positive psychology; place and well-being | X | X | |  | X |  |  |  | X |  |  |
| Foley | 2010 | Holy well; health and place; therapeutic landscape | X |  | | X | X |  |  | X | X |  |  |
| Wakefield and Mcmullan | 2005 | Health and place; therapeutic landscapes | X |  | |  |  |  |  | X | X |  |  |
| Zhou et al. | 2017 | Aging population; traveling for health enhancement; therapeutic landscapes; physical health; mental health | X | X | | X |  |  |  |  | X |  |  |
| Adongo et al. | 2017 | Spa; therapeutic activities; therapeutic landscape | X |  | | X |  | X |  | X | X |  |  |
| English et al. | 2008 | Role of place in health, healing, and recovery; breast cancer survivors; therapeutic landscapes | X | X | | X |  | X |  | X | X |  |  |
| Liamputtong and Suwankhong | 2015 |  | X | X | | X |  | X |  | X | X |  |  |
| Kaspar et al. | 2019 | Therapeutic mobilities | X |  | |  |  |  | X | X | X |  |  |
| Foley and Kistemann | 2015 | Geographies; blue space; ocean, coast, river, and lakes; health and place | X | X | |  |  |  |  |  | X |  |  |
| Smith | 2015 | Health tourism; health places; healing and recovery places |  |  | |  |  | X | X | X | X |  |  |
| Majeed et al. | 2018 | Medical tourism; wellness tourism; health resort; healing and recovery places | X | X | | X |  | X | X | X | X |  |  |
| Majeed et al. | 2017 | Medical tourism; mobility to therapeutic places; place-based wellness treatment | X | X | | X |  | X | X | X | X |  |  |
| Völker and Kistemann | 2011 | Blue space; air, water; and green scenery; therapeutic landscape; human cognition | X | X | | X | X |  |  | X | X |  |  |
| Jeuring and Becken | 2013 | Tourists and environment; protective behavior decisions; destination challenges | X | X | |  | X |  |  | X | X |  | X |
| Wang et al. | 2018 | Therapeutic space; sand therapy; disease, place, and health | X | X | |  |  |  |  |  | X |  |  |
| Majeed and Lu | 2017 | Medical tourism; preference | X |  | |  | X |  |  |  | X |  |  |
| Miller | 2007 | Disaster; hurricane; landscape; tourism |  |  | |  | X |  |  |  |  |  | X |
| Plzáková and Stupková | 2019 | Natural environment; health and well-being; visit to tourism destination | X | X | |  |  |  |  | X | X |  |  |
| Foley | 2013 | Holy well; place and health; well-being | X |  | | X |  | X |  | X | X |  |  |
| Elsubbaugh et al. | 2004 | Crisis management | X |  | |  |  |  |  | X |  |  | X |
| Dryglas and Salamaga | 2018 | Health tourism; spa resorts | X | X | | X |  | X |  | X | X |  |  |
| Majeed et al. | 2019 | Health enhancement; quality of life enhancement; well-being | X | X | | X |  | X |  | X | X |  |  |
| Majeed et al. | 2020 | Cosmetic surgery tourism; wellness tourism; medical tourism | X | X | | X | X | X | X | X | X |  |  |
| Laws and Prideaux | 2005 | Tourism; place and health; destination crisis | X |  | |  | X |  |  | X | X |  | X |
| Law | 2006 | Perceived risk; travel decisions | X |  | |  |  |  |  | X | X | X | X |
| Dolnicar | 2007 | Crisis; impacts on tourism | X |  | |  |  |  |  | X | X |  | X |
| Larsen et al. | 2009 | Perceived risks; tourist behaviors | X |  | |  |  |  |  | X | X | X | X |
| Wang et al. | 2020 | Sustainability; psychological well-being; nature and health | X | X | |  | X | X |  | X | X | X |  |
| Beirman | 2003 | Tourism; destination crisis; health crisis; health and place | X | X | | X | X |  |  | X | X |  | X |
| Pavli et al. | 2014 | Health crisis, MERs-Cov; travel precautions |  |  | |  |  | X |  | X | X |  | X |
| Pine and Mckercher | 2004 | Tourism; disease outbreak; SARS; Hong Kong’s tourism industry | X |  | |  | X |  |  | X | X |  | X |
| Tsi and Chen | 2010 | Tourism; earthquake | X |  | |  | X |  |  | X | X |  | X |
| Rodiek and Fried | 2005 | Landscape design | X |  | |  | X |  |  | X |  |  |  |
| Sonuç | 2020 | Wellness tourism; well-being; sustainability | X | X | | X | X | X |  | X | X |  |  |
| Seymour and Moore | 2000 | Crisis management | X |  | |  |  |  |  | X |  |  | X |
| Townsend et al. | 2018 | Therapeutic landscape; restorative environment; place attachment and well-being | X | X | | X | X |  |  | X | X |  |  |
| Ramkissoon et al. | 2018 | Natural environment; quality of life | X | X | |  | X |  |  | X | X |  |  |
| Ramkissoon et al. | 2013b | Place attachment; pro-environmental behavior | X | X | |  | X |  |  | X | X | X |  |
| Ramkissoon et al. | 2013a |  | X | X | |  | X |  |  | X | X | X |  |
| Ramkissoon et al. | 2012 | Place attachment; pro-environmental behavior | X | X | |  | X |  |  | X | X | X |  |
| Romao et al. | 2016 | Tourism growth; beach disease and consequence | X | X | |  |  |  |  | X | X |  | X |
| Wilder-Smith | 2006 | SARS; impact on travel and tourism | X |  | |  |  | X |  | X | X |  | X |
| Baker | 2015 | Tourism and health; impact of infectious disease and well-being | X | X | |  |  |  |  | X | X |  | X |
| Hassan and Ramkissoon | 2020 | Tourism; health benefits | X | X | |  |  |  |  |  | X |  | X |
| Hansen et al. | 2017 | Forest bathing; nature and therapy | X | X | | X | X |  |  | X | X | X |  |
| Brown and Raymond | 2007 | Place attachment; landscape values | X | X | | X | X | X |  | X | X | X |  |
| Ramkissoon and Mavondo | 2015 | Satisfaction and place; place attachment | X | X | | X | X | X |  | X | X | X |  |
| Mao et al. | 2010 | SARS, crisis management; tourist re-visits |  |  | |  |  | X |  | X | X |  | X |
| Rittichainuwat and Chakraborty | 2012 | Perceptions; destination crisis; tourism; destination management | X |  | |  |  |  |  | X | X | X | X |
| Aro et al. | 2009 | Travel; health risks; Influenza outbreak and tourists |  |  | |  |  |  | X | X |  | X | X |
|  |  |  |  |  | |  |  |  |  |  |  |  |  |
| Ramkissoon | 2016 | Island destination; place satisfaction, place attachment; well-being | X | X | | X | X | X |  | X | X | X |  |
| Ramkissoon and Mavondo | 2017 | Proenvironmental behavior; place attachment; satisfaction | X | X | | X | X |  |  | X | X | X |  |

JA = Journal Article; BK = book; BC= book chapter; PGTL = Perceived goodness of therapeutic landscapes; NL = natural landscapes; BL= built landscapes; LD = landscape design; HM= holistic medicine; TN = therapeutic networks; VDTL = visits to destination’s therapeutic landscapes; HWB = health and well-being; PA=place attachment; DC=destination crises
